# Gluten quality of bread wheat is associated with activity of RabD GTPases

**DOI:** 10.1111/pbi.12231

**Published:** 2014-07-22

**Authors:** Adam M Tyler, Dhan G Bhandari, Mervin Poole, Johnathan A Napier, Huw D Jones, Chungui Lu, Grantley W Lycett

**Affiliations:** 1 University of NottinghamLoughborough, UK; 2 Campden BRIChipping Campden, UK; 3 Rothamsted ResearchHarpenden, UK

**Keywords:** *Triticum aestivum*, endosperm, Rab GTPase, trafficking, gluten, breadmaking

## Abstract

In the developing endosperm of bread wheat (*Triticum aestivum*), seed storage proteins are produced on the rough endoplasmic reticulum (ER) and transported to protein bodies, specialized vacuoles for the storage of protein. The functionally important gluten proteins of wheat are transported by two distinct routes to the protein bodies where they are stored: vesicles that bud directly off the ER and transport through the Golgi. However, little is known about the processing of glutenin and gliadin proteins during these steps or the possible impact on their properties. In plants, the RabD GTPases mediate ER-to-Golgi vesicle transport. Available sequence information for Rab GTPases in *Arabidopsis*, rice, *Brachypodium* and bread wheat was compiled and compared to identify wheat RabD orthologs. Partial genetic sequences were assembled using the first draft of the Chinese Spring wheat genome. A suitable candidate gene from the *RabD* clade (*TaRabD2a*) was chosen for down-regulation by RNA interference (RNAi), and an RNAi construct was used to transform wheat plants. All four available *RabD* genes were shown by qRT-PCR to be down-regulated in the transgenic developing endosperm. The transgenic grain was found to produce flour with significantly altered processing properties when measured by farinograph and extensograph. SE-HPLC found that a smaller proportion of HMW-GS and large proportion of LMW-GS are incorporated into the glutenin macropolymer in the transgenic dough. Lower protein content but a similar protein profile on SDS-PAGE was seen in the transgenic grain.

## Introduction

Different Rab GTPases are involved in regulation fusion of transport vesicles with their different target membranes. In plants, certain types have expanded in number compared with yeast and mammalian systems, and in some classes, members may have diverged somewhat in function (Lunn *et al*., 2013; Lycett, 2008; Pereira-Leal and Seabra, 2001; Rutherford and Moore, 2002). The RabD clade is involved in ER-to-Golgi trafficking (Batoko *et al*., 2000; Cheung *et al*., 2002; Zheng *et al*., 2005), and in *Arabidopsis*, dominant negative mutant forms of RabD cause accumulation of secreted proteins and Golgi-resident proteins in an ER-like reticulate compartment (Batoko *et al*., 2000). However, in leaf protoplasts of other plant species, dominant negative RabD mutant proteins do not stop accumulation of storage proteins in protein bodies (Park *et al*., 2004), suggesting that RabD does not control all possible routes to the protein bodies.

The major wheat gluten proteins comprise two classes of prolamins, the monomeric gliadins and the glutenins that have interchain disulphide bonds that enable a complex polymeric structure to form (Kreis *et al*., 1985). The glutenins are classified into high molecular weight glutenin subunits (HMW-GS) and low molecular weight ones (LMW-GS), whereas the gliadins are classified by acidic polyacrylamide gel fractionation into α, β, γ and ω subunits (Woychik *et al*., 1961). Although the situation is complex, it seems that the elasticity of dough is conferred mainly by the glutenins and the extensibility mainly by the gliadins (Shewry *et al*., 1995). Good breadmaking wheat is characterized by a high protein content, but the type of protein is also important.

Gluten proteins are synthesized on the rough endoplasmic reticulum (RER) where proteins may be folded, disulphide-bonded and hydrogen-bonded. This can cause them to precipitate to form hydrated protein particles (Tosi *et al*., 2009). Whether different proteins are trafficked at different times and whether they go by different routes from the ER to the protein bodies is still uncertain. Rubin *et al*. (1992) proposed that one type of protein body, containing large insoluble HMW protein aggregates, buds directly from the ER, whereas less dense bodies form from protein trafficked in transport vesicles via the Golgi apparatus. An attempt to confirm this by the use of specific antibodies for α, β, γ and ω gliadins, and HMW and LMW glutenin subunits failed to showed any difference in the protein content of storage vesicles (Loussert *et al*., 2008), but protein sorting into different protein bodies was shown by Tosi *et al*. (2009) using a very specific antibody for a LMW glutenin subunit. Golgi trafficking is favoured during the early grain-filling stage, whereas aggregation in the ER occurs at later stages and Golgi-stacked cisternae are no longer visible after 12 days postanthesis (dpa) (Levanony *et al*., 1992; Loussert *et al*., 2008; Tosi *et al*., 2009).

Biotechnological attempts to improve gluten quality have mainly focused on expression of specific proteins, especially the HMW glutenins (Leon *et al*., 2009, 2010; Rakszegi *et al*., 2008; Tatham *et al*., 1990). However, the use of a dominant negative mutant tobacco *RabD* transgene improved gluten strength and increased disulphide bonding in tetraploid durum wheat (Di Luccia *et al*., 2005). In this study, we have used RNAi to knock down the expression of RabD GTPase genes in the hexaploid breadmaking wheat variety Cadenza and have analysed the effect on dough quality.

## Results

### Bioinformatics

**Figure 1:**
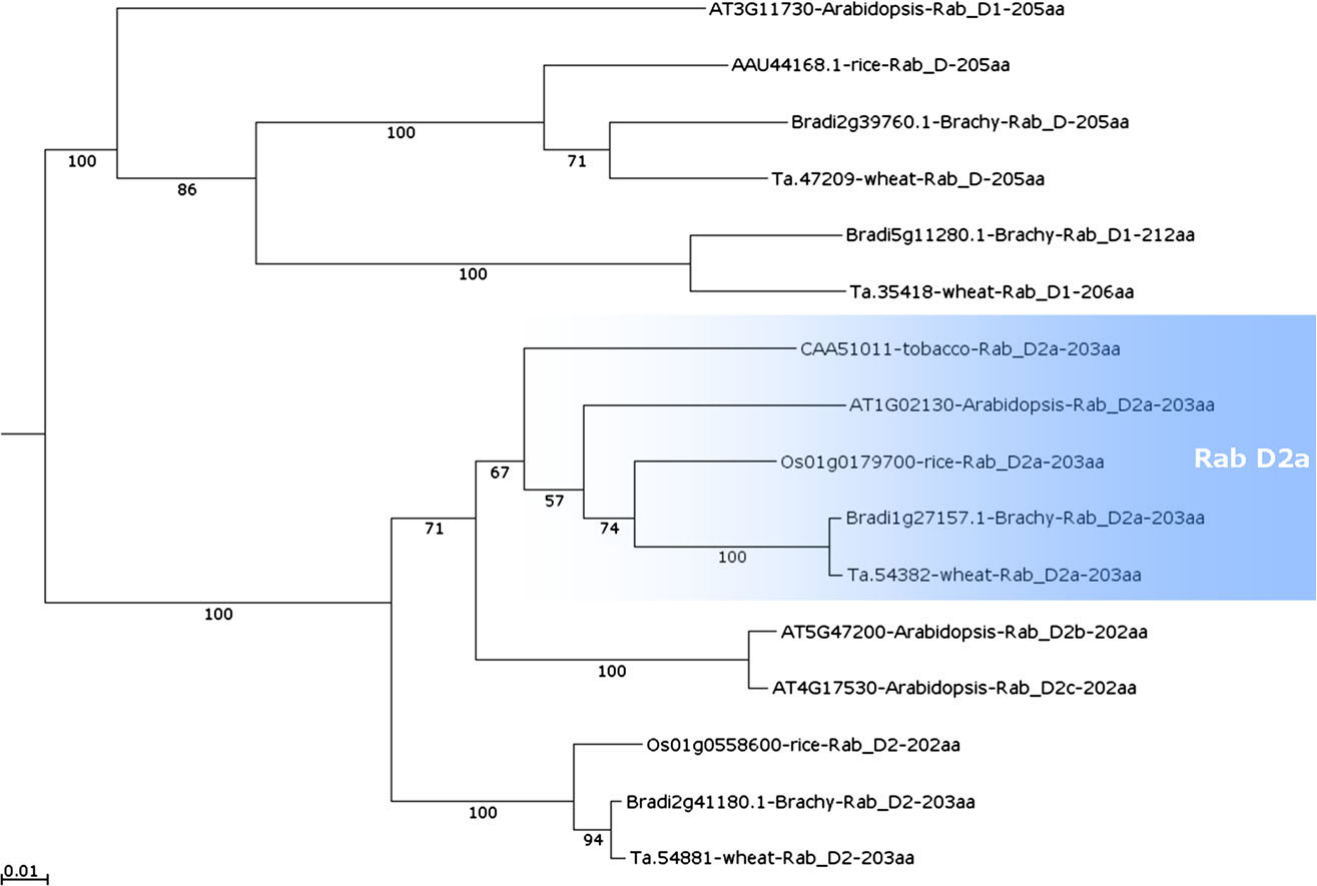
Phenogram with Rab D protein sequences of wheat, *Brachypodium*, rice, *Arabidopsis* and tobacco. Rab D2a homologs are highlighted in blue. Alignment and phenetic tree file were generated using MAFFT version 6 online (Katoh and Toh, 2010). Tree is neighbour-joining with 1000 bootstrap replicates, shown on branches as percentage confidence values.

Phenetic analyses were carried out using Rab protein and nucleotide sequence data from *Arabidopsis thaliana*, *Brachypodium distachyon*, rice, wheat and tobacco. Selected sequences were aligned, and a phenetic tree was generated to discern relationships between all RabD proteins from the four organisms (Figure1).

**Figure 2:**
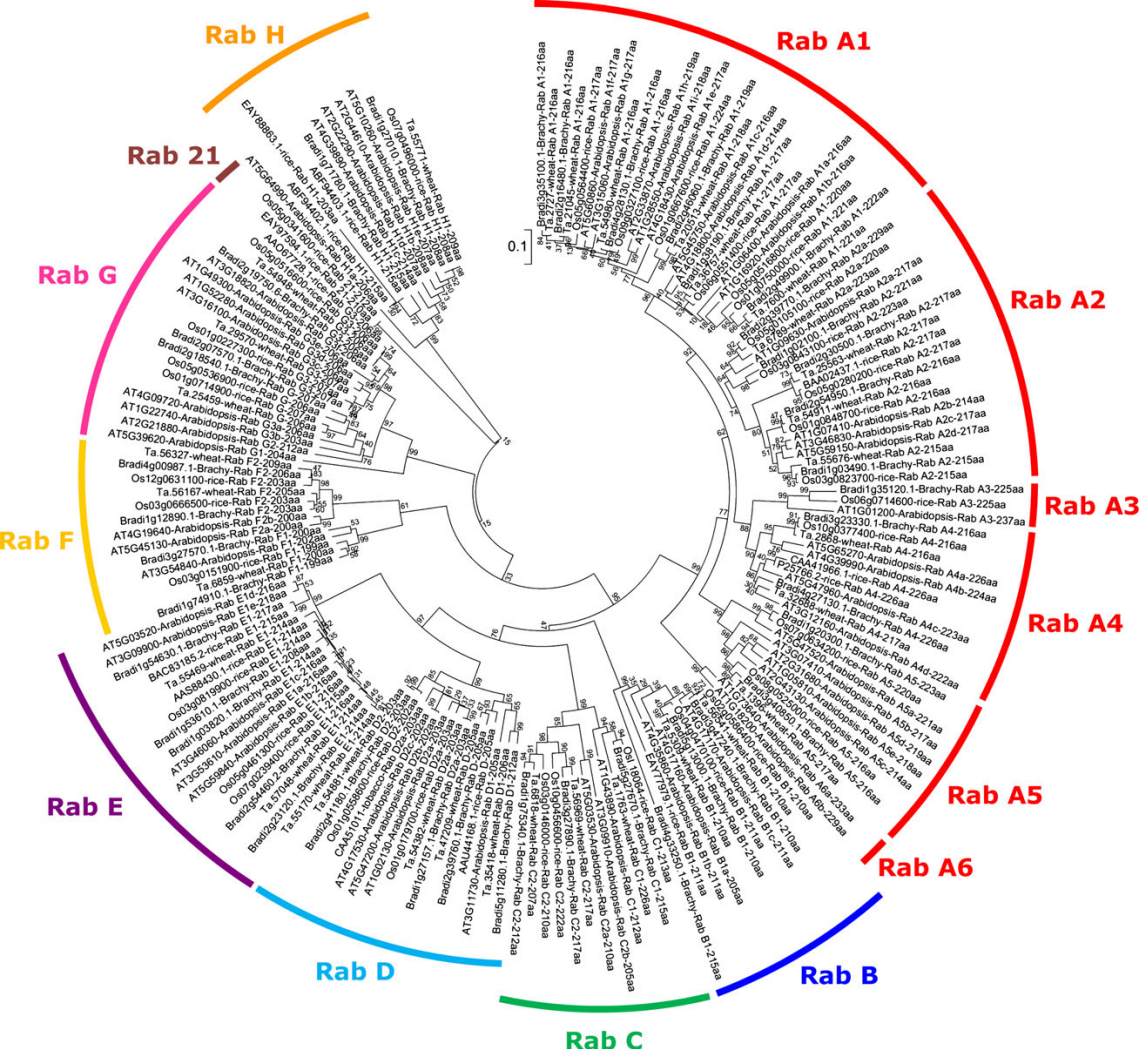
Circular phenogram containing 175 Rab protein sequences from *Arabidopsis*, rice, *Brachypodium* and wheat. Rab clades indicated on the outside. Tree is neighbour-joining with 1000 bootstrap replicates, shown on branches as percentage confidence values. Branches corresponding to partitions reproduced in <50% bootstrap replicates are collapsed.

A cladogram comparing all Rab proteins identified (Figure2) proved helpful in the more specific identification of Rabs in *Brachypodium* and wheat. The Rab protein family is conserved within clades rather than within species, demonstrating their equivalent function in the two different plants.

### Production, selection and screening of transgenic wheat lines

For the production of transgenic plants, we chose the variety Cadenza, which is a facultative winter variety with moderate breadmaking quality, rated 6 on a 1–9 scale where 9 is highest quality (Rakszegi *et al*., 2008). Wheat plants were transformed by biolistics with an RNAi construct cloned into the pHMW-Adh-Nos vector (Nemeth *et al*., 2010; Figure S1) to give endosperm-specific expression. This construct targeted a 270-nucleotide section of the Ta.54382 (RabD2a) coding sequence that included a 23nt match with Ta.54881 (RabD2b). All 29 T0 plants, designated L1 to L29, were screened for transgene presence using PCR. Based on PCR results, 16 T0 plants were selected, each of which was an independent transformation event. From these 16 selected T0 plants, 24 seeds from each plant were initially sown, of which 12 from each plant were grown on to maturity. The designation for each of these mature T1 plants consists of the name of the parent plant followed by .1 to .12. Probable zygosity of all 192 T1 plants was determined by PCR of T2 seedlings for transgene presence. T2 grains from lines that had sufficient grain weight for rheological testing were analysed for size and shape, protein content, moisture content and dough-mixing properties, along with controls. A homozygous transgenic line (L10.5) that displayed a large difference in mixing properties yet similar protein and moisture content compared with the controls was selected to be grown alongside a control line (L24.2).

### Expression of *RabD* genes in transgenic lines

To establish that the target *RabD* gene (Ta.54382) had been successfully knocked down and to check whether other *RabD* genes with close sequence similarity were also affected, a quantitative real-time PCR was performed looking at the expression of four *RabD* genes in the endosperm of T3 lines L10.5 (knock-down) and L24.2 (control) at 14 and 21 dpa.

Three other genes—*Ta.2291*, *Ta.2776* and *Ta.54227—*were also included as potential normalization genes based on a study identifying reference genes in wheat (Paolacci *et al*., 2009). However, expression of *Ta.2291* appeared to be 79% higher at 14 dpa and 120% higher at 21 dpa in the transgenic line compared with the control and was therefore not used as a normalization gene in this study. qRT-PCR data of the *RabD* genes were normalized using the *Ta.54227* gene (for primers see Tables 1 and 2). Calibration curves were set up for each primer pair using serial dilutions of a sample containing a mixture of all cDNA samples.

**Table 1:** Primers used in qPCR

Unigene	Primer F name	Primer F sequence	Primer R name	Primer R sequence
Ta.35418	Ta.35418-RT1-F	TCC GTT TCT CCG ACG ATT CG	Ta.35418-RT1-R	TCC TGC TGT GTC CCA AAT CTG
Ta.47209	Ta.47209-RT4-F	AGG TCG TCG ATA CAG AGG AG	Ta.47209-RT4-R	GGC TCG CCA TCT TGT TCT TG
Ta.54832	Ta.54832-RT3-F	ATC GGA GAC TCA GGT GTT GG	Ta.54832-RT3-R	GTT CTG AAG CGT TCT TGC CC
Ta.54881	Ta.54881-RT3-F	AAG TCT TGC CTG CTG CTG AG	Ta.54881-RT2-R	TCG CTC TTG TCC AGC AGT ATC
Ta.2291	Ta.2291-P1-F	GCT CTC CAA CAA CAT TGC CAA C	Ta.2291-P1-R	GCT TCT GCC TGT CAC ATA CGC
Ta.2776	Ta.2776-P1-F	CGA TTC AGA GCA GCG TAT TGT TG	Ta.2776-P1-R	AGT TGG TCG GGT CTC TTC TAA ATG
Ta.54227	Ta.54227-P1-F	CAA ATA CGC CAT CAG GGA GAA CAT C	Ta.54227-P1-R	CGC TGC CGA AAC CAC GAG AC

Sequences are 5′-3′**.**

**Table 2:** Fragments amplified by PCR

Unigene	Putative *Rab* name	cDNA product/bp	gDNA product/bp
Ta.35418	*RabD1a*	122	>122
Ta.47209	*RabD1b*	141	547/866
Ta.54832	*RabD2a*	176	1176
Ta.54881	*RabD2b*	147	1517

**Figure 3:**
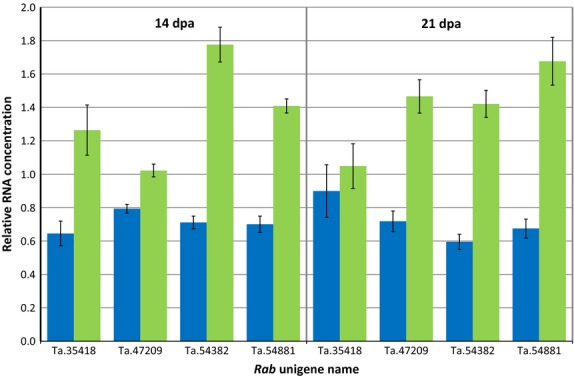
Expression of *RabD* genes at 14 and 21 days postanthesis in the developing endosperm of T3 wheat. Blue bars: L10.5 (GM), green bars: L24.2 (control). Expression normalized with Ta.54227 gene (cell division control). Error bars indicate standard error. *RabD1a* = Ta.35418; *RabD1b* = Ta.47209; *RabD2a* = Ta.54382; *RabD2b* = Ta.54881.

All four *RabD* genes were found to some extent to have lower transcript levels at 14 dpa in the transgenic line compared with the control (Figure3). The *RabD1* genes Ta.35418 and Ta.47209 had 49% and 22% reductions in transcript levels, respectively. The expression of *RabD2a* target gene *Ta.54382* and its closest relative gene *Ta.54881* showed a 60% and 50% reduction, respectively, compared with the control. At 21 dpa, the transcript levels of *RabD2* genes *Ta.54382* and *Ta.54881* again showed similar reductions in the transgenic line compared with the control line of 58% and 60%, respectively. However, at 21 dpa, whereas *Ta.47209* transcript levels were 51% lower in the RNAi line than the control, there was no significant difference in the *Ta.35418* transcript levels in the two lines. The transgene clearly had a large effect on expression of *RabD* genes in the developing endosperm, with *RabD2* genes down-regulated by 50%–60% at both 14 and 21 dpa.

### Size and shape analysis of transgenic grain

To investigate potential effects of the transgene on grain dimensions and proportions, an analysis of the size and shape of the T2 grain was performed. Using DigiEye apparatus, photographs of grain belonging to 41 lines were taken, including six lines from the L24 bar-only control parent. The photographs were analysed with ImageJ software to give values for area, perimeter, length, width and circularity of each grain image. The number of grains analysed for each line ranged from 76 to 703 but was mostly in the range 150–200. The mean of each of the values was determined for each line. While L05.6 apparently had the highest mean perimeter and width, it had the lowest number of grains analysed (76); therefore, its mean values are the least reliable (Figure S2). The control lines tend towards the mid range in most of the properties, with a slight leaning towards the higher range in area and the lower range in perimeter, and with a wide spread in length. This implies that the transgene has little effect on these grain properties. However, the control lines appeared to have greater circularity than most of the other lines, which would suggest that the transgene reduces grain circularity (i.e. causes a more elongated grain shape).

To determine whether differences seen in the T2 generation were also present between lines of T3 grain, a similar analysis was performed on T3 grain from the selected T2 knock-down line L10.5 and a T2 control line L24.2. The data set for L10.5 was produced through analysis of 1329 grains and that for L24.2 from 984 grains. There is a slightly greater perimeter and smaller area in the grains of the T3 knock-down line compared with the control line (Figure S3). Additionally, the knock-down grains are slightly longer and thinner. These combined small differences result in a significant reduction in circularity of the knock-down line compared with the control. This finding agrees with the results from the previous generation, which further suggests that the changes seen are caused by the transgene.

### Protein content of grain

After milling, protein content of the T2 flour was measured to compare the total levels of protein in the 20 selected lines. The mean protein content of the control T2 lines on a dry weight basis was 16.38%, while that of the T2 lines found to contain the transgene was 14.83% (Figure S4). This drop in mean protein content of 1.55% in the transgenic lines was despite the anomalously high protein level of transgenic line L03.11.

**Figure 4:**
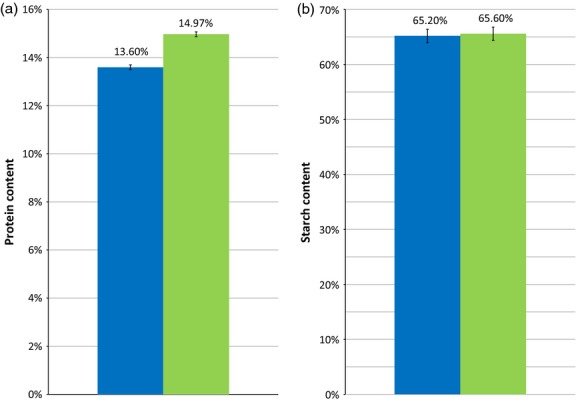
Protein and starch content of flour from knockdown and control T3 lines as a percentage of dry weight. (a): Protein content of T3 lines on dry weight basis; (b): starch content of T3 lines. The blue bars represent T3 knockdown line L10.5, the green bars represent T3 bar-only control line L24.2. Error bars indicate predicted error of measuring techniques.

There was also a 1.37% reduction in total protein in the T3 transgenic line L10.5 compared with the control line L24.2 (Figure4a). This finding concurs with the differences in protein content of T2 transgenic lines compared with the T2 controls and specifically the difference between T2 lines L10.5 and L24.2, which indicates the difference is a result of the transgene. No significant difference in starch content was found between the T3 knock-down line and control line (Figure4b).

### SDS-PAGE analysis of mature wheat seed protein

**Figure 5:**
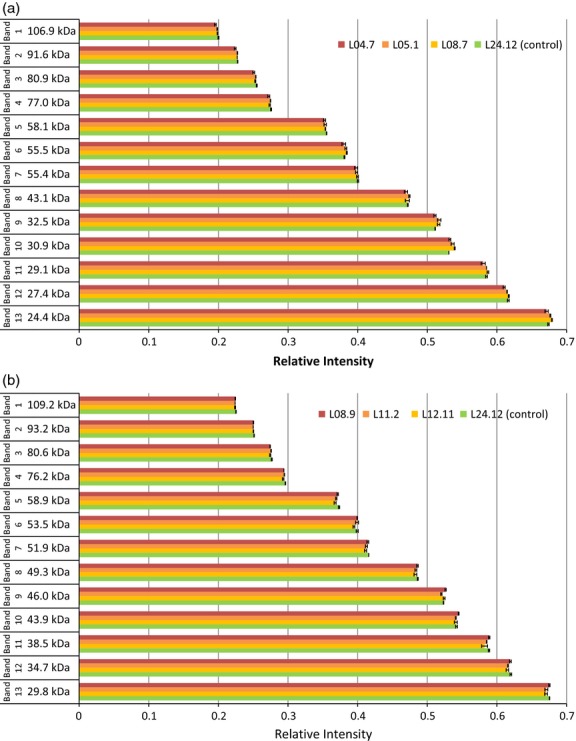
Examples of densitometry analysis of T2 SDS-PAGE. (a) Densitometry analysis of Figure S5f and (b) densitometry analysis of Figure S5g. The molecular weights of 13 bands are indicated on the left in kDa. Error bars indicate standard error.

To better understand protein deposition in the transgenic grain, SDS-PAGE experiments were carried out on mature seeds of T0 plants (Figure S5) and the mature seeds of 30 lines selected by PCR and grain weight (Figure S6). Controls used included grain from bar-only and gold-only T0 plants and from a bar-only T1 plant. Densitometry was performed by the Cereals and Milling Department at Campden BRI on 5 of the gels, each comparing 2–3 transgenic lines with control line L24.12 in triplicate. Only very small differences were found between the intensity of the bands in transgenic lines compared with the controls (Figure5). This suggests that the transgene has little, if any, effect on the ratio of different seed storage proteins.

### Dough rheology

The main factor in deciding which homozygous T2 lines to grow on in the next generation was a study of the processing properties of the dough. Twenty lines were chosen to be milled, based on transgene PCR confirmation and grain weight. Milling was carried out in a laboratory scale Quadrumat mill. Coarse semolina and bran particles were subjected to two passes through the milling process. The resulting flour samples were tested for moisture and protein content and mixed into dough using a Reomixer apparatus. The Reomixer produced traces of resistance to mixing over the course of 10 min, measured in arbitrary units of torque from 0 to 10. The important features of the trace are peak torque, dough development time (time at peak torque), breakdown amount/angle (reduction in dough strength after peak torque) and trace width.

**Figure 6:**
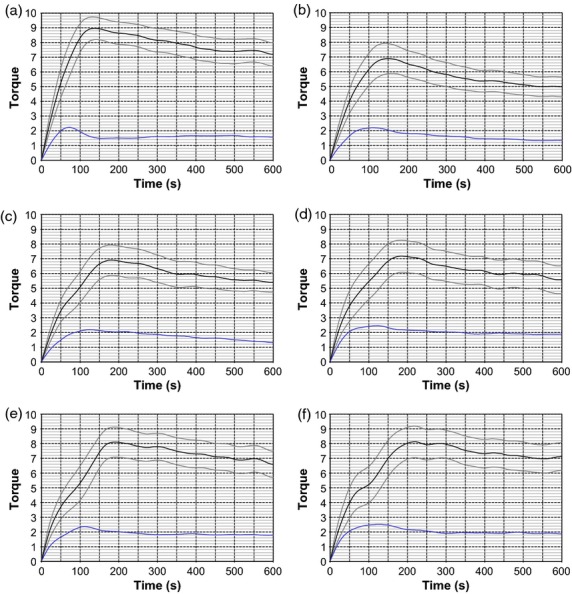
Six examples of torque traces during mixing of dough made with flour from T2 lines. (a) High protein line L25.1; (b) homozygous line L10.5; (c) homozygous line L12.11; (d) homozygous line L17.12; (e) control line L24.2; (f) control line L24.10. Black line indicates middle of trace, grey lines indicate edges of trace, and blue line indicates trace width. Torque values are relative.

The high protein line L25.1 showed a high peak torque of 9 but a thin trace and short development time of 130 s (Figure6a). On the other hand, homozygous T2 lines L10.5, L12.11 and L17.12 had substantially lower peak torques of around 7 but somewhat longer development times (150–190 s; Figure6b–d). Trace width varied between them – L17.12 had the widest trace (similar to the controls) and the trace of the other two lines appeared to gradually narrow after peak torque. The peak torque of the four control lines was in the range 8.1–8.4 and occurred after 195–225 s. Trace width was greater than most of the transgenic lines.

**Figure 7:**
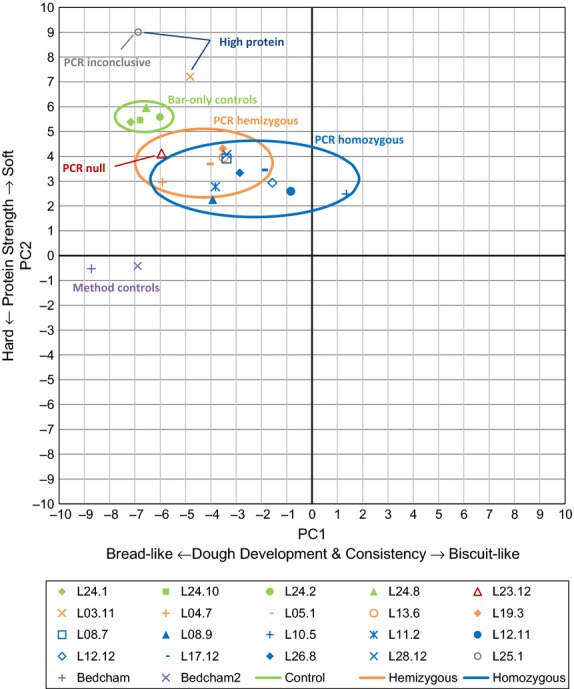
Quality map of T2 flour reomixer data. Green points: bar-only control lines; orange points: appear hemizygous; blue points: appear homozygous; purple points: method control; red point: appears null segregant; and grey point: zygosity unknown. Ellipses are calculated from a group of coordinates based on genotype (control, hemizygous or homozygous) at ±2.45 standard deviation from the mean. The high protein line L03.11 was not included in the ellipse calculations

From the traces of the twenty lines, variables were fed into two dough quality indicators—PC1 and PC2, as defined by Anderson (2004). PC1 increases as peak torque and dough development time decrease; therefore, weak biscuit-like flours have a higher PC1 and strong bread-like flours give a lower PC1. PC2 is determined by breakdown—greater breakdown gives a higher PC2 value—so a more stable dough will have a lower PC2. The PC values were calculated for each line and plotted on a quality map (Figure7).

Excluding the anomalous high protein line L03.11, transgenic T2 lines had higher PC1 values and lower PC2 values than the controls, and homozygous lines more so than hemizygous lines. A null segregant included in the experiment showed similar PC1 to the controls but somewhat decreased PC2, although only one hemizygous line had a PC2 value between the null segregant and the control lines. These results strongly suggest that presence of the transgene significantly altered the processing properties of the dough.

The small-scale rheology results from the T2 generation showed substantial changes in the processing characteristics of the dough of transgenic lines. To demonstrate the specific changes in gluten quality on a larger scale, farinograph and extensograph tests were carried out on T3 dough. While similar to the Reomixer test performed on T2 flour, the farinograph is a more conventional method of determining dough processing properties.

**Figure 8:**
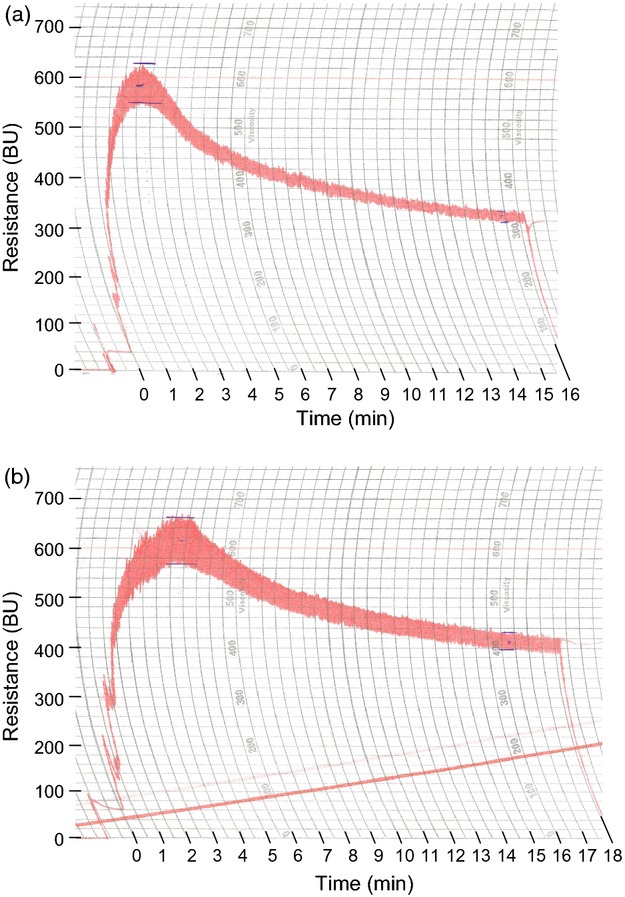
Farinograph traces of dough made with flour from T3 lines. (a) T3 knock-down line L10.5 (development time 2 min, stability 1.5 min, degree of softening 250BU); (b) T3 bar-only control line L24.2 (development time 3.5 min, stability 2.5 min, degree of softening 200BU). The farinograph measures and records the resistance of dough to mixing as it is formed from flour and water, is developed and is broken down.

In the farinograph traces, there are clear differences between the T3 knock-down line L10.5 and the T3 control line L24.2 (Table 4, Figure8). The dough development time (time until peak resistance) was noticeably shorter in the knock-down line and the dough started to break down more quickly after peak resistance. Additionally, the width of the trace in the knock-down line was smaller than in the control throughout the mixing, and the resistance dropped to a lower level compared with the control. The specific measurements made by the farinograph back up these observations. The knock-down line had a 43% reduction in development time, 40% reduction in stability (time during which the top edge of the trace is above the centre of the peak resistance) and 25% increase in degree of softening (difference in resistance between peak resistance and 12 min after peak resistance) compared with the control line.

**Table 3:** Primers designed to anneal to the target sequence in PCR

Primer name	Sequence 5′-3′
−site primer F	GTTGGCAAGTCATGCCTTC
−site primer R	GCGATCAATCTCGTTCAACC
+site primer F	*CTTAA* **GGATCC** GTTGGCAAGTCATGC
+site primer R	*GGCCA* **AGATCT** GCGATCAATCTCGTT

The parts of the +site primers that match the gene (underlined) are preceded by a restriction site (bold), plus extra nucleotides to ensure efficient restriction (italics). +site primer F contains a *Bam*HI site, and +site primer R contains a *Bgl*II site.

**Table 4:** Farinograph and extensograph results

	Control line L24.2	Transgenic line L10.5
Farinograph features		
Development time (min)*	3.5	2.0
Stability (min)†	2.5	1.5
Degree of softening (BU)‡	200	250
Extensograph features		
Resistance (BU)§	210	120
Extensibility (cm)¶	23.5	16.0

* Development time refers to time until peak resistance.

† Stability is the time during which the top edge of the farinograph trace is above the centre of the peak resistance.

‡ Degree of softening is the difference in resistance between peak resistance and 12 min after peak resistance in Brabender units (BU).

§ The extensograph resistance value refers to maximum resistance in Brabender units (BU).

¶ Extensibility refers to the distance stretched before breaking.

**Figure 9:**
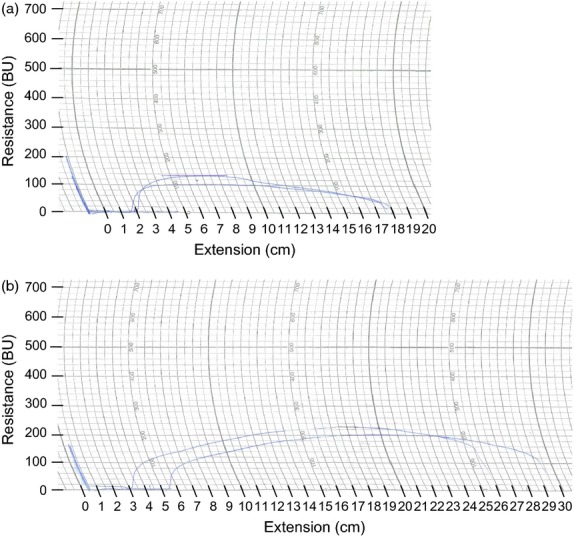
Extensograph traces of dough made with flour from T3 lines. (a) T3 knock-down line L10.5 (resistance 120BU, extensibility 16 cm); (b) T3 bar-only control line L24.2 (resistance 210BU, extensibility 23.5 cm). The dough mixed by the farinograph is moulded on the extensograph into a standard shape and rested for 45 min before being stretched to breaking point on the extensograph and a curve drawn recording the extensibility of the dough and its resistance to stretching. The dough is immediately remoulded and, after a further rest period, is re-stretched.

The extensograph traces and corresponding values show that dough made from the T3 knock-down line was weaker and less extensible than the control line (Table 4, Figure9). This is reflected by the resistance and extensibility figures, which were, respectively, 43% lower and 32% lower in the knock-down line compared with the control.

### SE-HPLC analysis of gluten macropolymer

As the difference in protein content was relatively small compared with the alteration of dough rheology in the T2 generation and the relative levels of gluten subunits were very similar (as shown by SDS-PAGE results), it was likely that other factors were contributing to the processing properties of the dough. To investigate the characteristics of the large disulphide-linked aggregates formed by polymeric subunits in the glutenin macropolymer that gives dough its elastic properties, a size exclusion high-performance liquid chromatography (SE-HPLC) experiment was carried out using T3 flour. The largest polymeric gluten proteins, high molecular weight glutenin polymers, elute as a peak between 9m and 10m10s (fraction F1); the smaller polymeric gluten proteins, low molecular weight glutenin polymers, elute between 10m11s and 13m40s (fraction F2); the monomeric gliadin proteins elute between 13m41s and 14m45s and between 14m46s and 16m50s (fractions F3 and F4, respectively); and the globulin/albumin fraction elutes between 16m51s and 19m (fraction F5). Two replicates of each T3 line were run untreated (i.e. without sonication) and with sonication during extraction with SDS solution. Sonication conditions were selected to maximize the solubilization of the polymeric proteins, while minimizing the breakdown of the polymers and disruption of the native protein structures within the flour (Morel *et al*., 2000).

**Figure 10:**
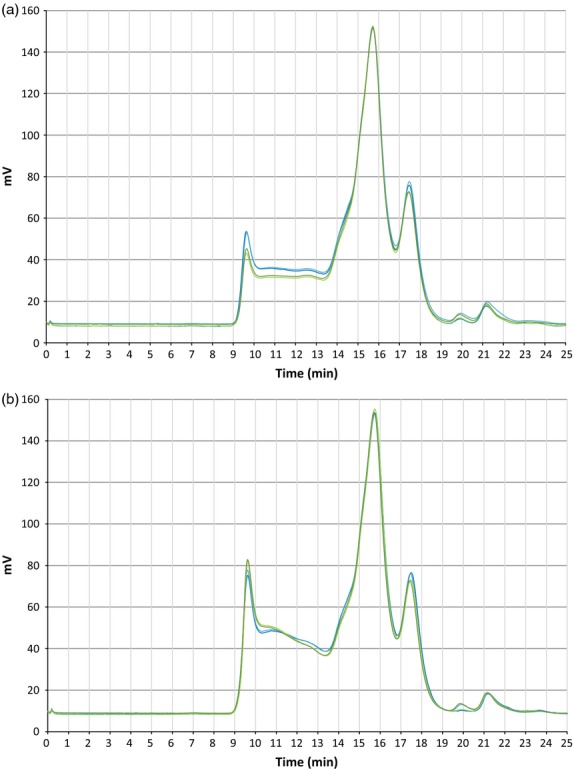
SE-HPLC traces of protein from T3 flour samples. (a) Untreated flour sample; (b) sonicated flour sample. The two replicates of T3 line L10.5 are in light and dark blue, and the two replicates of T3 bar-only control line L24.2 are in light and dark green. Proteins with a higher molecular weight migrate through the column faster and are on the left of the plot while smaller proteins take longer to migrate through the column and are on the right. The purpose of the sonication was to promote solubilization of the glutenin macropolymer. Proteins present in the untreated samples consist of monomers, oligomers and glutenin subunits, which were only weakly bound to the glutenin macropolymer. The sonicated samples should contain the same proteins as the equivalent untreated samples plus glutenin subunits that have been released from the macropolymer.

The SE-HPLC profiles of the untreated (not sonicated) samples show that a greater proportion of polymeric high molecular weight glutenin polymer and low molecular weight glutenin polymer proteins, in fractions F1 and F2, respectively, had been extracted in the T3 knock-down line L10.5 compared with the T3 control line L24.2 (Table 5, Figure10a). This suggests that the modified knock-down line featured a gluten macropolymer complex that was easily solubilized and hence was weaker than the control line. Examination of the sonicated samples showed that the relative size distribution of the polymeric proteins within the total extractable gluten protein fraction was different between the knock-down lines and the control lines (Table 5, Figure10b). Here, the knock-down line shows smaller peaks eluting in F1 and between 10m11s and 11m20s in F2 compared with the control line, indicating that it contained a lower proportion of the higher molecular weight polymeric gluten material. The presence of more, smaller low molecular weight glutenin polymer polymeric protein in the knock-down line, eluting between 11m21 and 13m40s in F2, suggests that it had been only weakly bound within the gluten macropolymer matrix, compared with the control line.

**Table 5:** SE-HPLC fractions

Line	Sonicated	SE-HPLC fraction*								

		F1	F2	F3	F4	F5	F1/F2	F3+F4/F1	(F3+F4)/(F1+F2)	AT†
Transgenic L10.5	Yes	11.9	23.0	8.4	41.1	15.5	0.52	4.14	1.42	31.5
Control L24.2	Yes	12.9	23.0	8.0	41.3	14.8	0.56	3.83	1.37	30.9
Transgenic L10.5	No	8.7	18.8	8.9	45.9	17.7	0.46	6.30	1.99	27.7
Control L24.2	No	7.5	17.3	8.9	48.5	17.7	0.43	7.67	2.31	25.8

* F1–F5 values represent the area under the curve for each of the fractions defined by the Profilblé method that were separated by SE-HPLC. These fractions relate to different protein subunits extracted from the gluten macropolymer.

† Calculations of the fractions and the total area under the curve (AT) are also presented.

## Discussion

Transcript levels of all four wheat *RabD* genes studied were reduced in the transgenic line at 14 dpa, and all but Ta.35418 (*RabD1*) were knocked down at 21 dpa. The transgenic effect is a result of a pHMW-Adh-Nos RNAi vector containing a 270-bp sequence taken from a single *Rab* gene, Ta.54382 (*RabD2a*) with a 21nt match with Ta.54881 (*RabD2b*). A recent study by Wang *et al*. (2012) found that overexpression of a YFP-associated mutant *RabD2b* gene in *Arabidopsis* led to co-suppression of the native *RabD2b* gene along with down-regulation of *RabD1 and RabD2c* genes, although *RabD2a* appeared unaffected. A similar down-regulation mechanism might help account for the expression pattern seen in the developing T3 seeds, where aside from the target gene, three other genes of the same subfamily showed reduced transcript levels. Alternatively, the fact that this strategy appeared to affect a number of similar genes regardless of whether or not there was a perfect 21nt match between them could be due to cleavage of mRNA molecules with sequences that are highly conserved with the siRNA incorporated into RISC, in addition to those that are fully identical as suggested by a number of recent studies (Birmingham *et al*., 2006; Fedorov *et al*., 2006; Haley and Zamore, 2004; Sigoillot *et al*., 2012).

A potential reference gene included in the qRT-PCR experiment, Ta.2291 (*Arf2*), which had previously been described as a suitable reference gene for qPCR in wheat (Paolacci *et al*., 2009), seemed to be unsuitable as a reference gene and in fact appeared to be significantly up-regulated in the transgenic line compared with the control at both 14 dpa and 21 dpa. This is an interesting result as Arf (ADP ribosylation factor) proteins are small GTPases involved in vesicle trafficking and are part of the same Ras superfamily as Rab GTPases (Wennerberg *et al*., 2005). It would appear that reduced *RabD* levels affected some equilibrium or feedback mechanism in the vesicle transport system, leading to increased expression of at least one *Arf* gene, perhaps to compensate for reduced ER-to-Golgi vesicle traffic.

The T3 rheology results clearly indicate a reduction in dough strength and extensibility in the knock-down line compared with the control. It is likely that altered intermolecular bonding in the glutenin macropolymer contributed to this effect, combined with a drop in total protein content. As it has been confirmed that the *RabD* genes are knocked down in the developing endosperm in the T3 transgenic line relative to the control line, it is reasonable to conclude that the expression of one or more of these genes is instrumental in gluten quality.

The SE-HPLC findings provide evidence that the *RabD* knock-down transgene has markedly affected interactions or bonding between glutenin subunits in the gluten macropolymer of dough. Bonding strength appears most reduced in high molecular weight glutenin polymer and some of the larger low molecular weight glutenin polymers. As these subunits are probably the most important in giving gluten its elastic properties (Anjum *et al*., 2007), the transgenic dough would be expected to have weaker gluten strength when processed. This correlates with the results of the rheology experiments, in which the knock-down lines exhibited considerable reductions in gluten strength and extensibility compared with controls. These changes are within the range of quality criteria for commercial cultivars. The reduction in gluten strength represents a transformation from bread processing suitability to characteristics which are more similar to biscuit processing suitability. However, the reduction in extensibility is likely to limit the suitability of this sample to specialist products other than bread or biscuit processing.

A number of studies on glycosylation of gluten proteins have yielded results indicating that a significant proportion of wheat storage proteins are glycosylated (Gujska and Khan, 1999; Lauriere *et al*., 1996; Tilley, 1997; Tilley *et al*., 1993). One study found that gliadins and glutenins were both glycosylated, and aggregated LMW-GS showed N-glycans with xylose, thought to indicate processing in the Golgi (Lauriere *et al*., 1996). Meanwhile, many other studies describe finding little or no glycosylation of HMW-GS and α-gliadins using mostly mass spectrometric approaches such as GC-MS, RP-HPLC/ESI-MS and MALDI-TOF-MS (Bollecker *et al*., 1998; Cunsolo *et al*., 2002, 2003, 2004; Foti *et al*., 2000; Roels and Delcour, 1996; Turner *et al*., 2002; Zhang *et al*., 2008). Additionally, some of these studies also discovered false-positive results when using other techniques including periodate-digoxigenin glycan detection assay and acid PAGE (Bollecker *et al*., 1998; Roels and Delcour, 1996). While evidence has built up that HMW-GS are probably not commonly glycosylated, LMW-GS and gliadins are less well studied, and there is still uncertainty whether they undergo glycosylation or not (Li *et al*., 2008). If any LMW-GS or gliadins are glycosylated, then it is likely that reducing ER-to-Golgi vesicle traffic would have a profound effect on this process. It is possible that a change in prolamin glycosylation could be at least partly responsible for differences seen in the transgenic wheat grain, although it is unclear whether or not this is the case.

SE-HPLC and rheology results show a tangible difference in dough quality between the transgenic and control lines. However, SDS-PAGE results suggest that the relative proportions of the different gluten subunits have not been affected. A significant contributing factor to this may be the likelihood that fewer gluten proteins in the transgenic lines than in the control lines go through important post-translational modifications that occur in the Golgi, due to inhibited ER-to-Golgi trafficking. It is likely that post-translational modifications have occurred in the Golgi that affect bonding ability of gluten proteins. These modifications could be in the form of disulphide bonding, hydrogen bonding, glycosylation or folding into a structure able to produce the loop and chain arrangement thought to give gluten its elasticity in dough (Belton, 1999). Protein disulphide isomerase (PDI) is located in the ER and protein bodies, and although this glycoprotein is thought to catalyse intermolecular covalent bonding between glutenin subunits, it is not known whether it has a role in folding of the subunits (Shimoni *et al*., 1995).

There is a general consensus that wheat prolamins are transported to the protein storage vacuole by two different pathways: Golgi dependent and Golgi independent (Tosi, 2012). However, there is disagreement as to whether or not different prolamins are transported through the two routes differentially, and if so, what causes the difference (Levanony *et al*., 1992; Loussert *et al*., 2008; Rubin *et al*., 1992; Tosi *et al*., 2009). Based on SE-HPLC results of this study, the reduction of *RabD* expression in wheat endosperm appears to reduce the bonding ability of HMW-GS and larger LMW-GS while smaller LMW-GS are less affected. This may point towards different glutenin subunits favouring different trafficking routes or alternatively that smaller glutenins are not as dependent on processing in the Golgi to retain their ability to bond in the glutenin macropolymer. However, if there is a difference in the ratio of proteins trafficked via the Golgi compared with ER aggregation, it is not apparent in the SDS-PAGE results, which do not show any differences in subunit proportions present in the mature seed between the knock-down and control lines. It is possible that proteins blocked by one route could, subsequently, be trafficked by the other route.

The knowledge gained from this study regarding the sequence and function of Rab GTPases in the endosperm of hexaploid wheat could be further exploited to better understand, and possibly improve, the technological properties of flour. Based on the results of this work, it would appear that *RabD* expression is linked to dough processing quality in bread wheat, at least in that gluten quality appears reduced when *RabD* expression is reduced. Conversely, it may therefore be inferred that an increased level of *RabD* expression might improve gluten quality. However, overexpression of native *RabD* genes has met with a lack of phenotype in studies on durum wheat and *Arabidopsis* (Di Luccia *et al*., 2005; Wang *et al*., 2012). When no phenotype was found in transgenic *Arabidopsis* overexpressing RAC/ROP small GTPases, the authors of the study suggested that interactors including GAPs and GDIs could be responsible for keeping GTPase activity stable despite a greater quantity of protein present (Brembu *et al*., 2005). It is reasonable to conclude that the wide range of Rab interaction partners could prove problematic to direct efforts to up-regulate vesicle trafficking from ER to Golgi by genetic modification.

Most attempts to alter breadmaking quality in bread wheat using biotechnology have targeted the gluten proteins themselves, particularly the HMW glutenin subunits. This study represents a novel approach to this area of work by altering the transport of these important proteins in the endosperm. The resulting effects on grain shape, protein content, bonding in the glutenin macropolymer and dough rheology reported here expand the understanding of factors that contribute towards gluten quality in bread wheat and could be of interest to breeders looking for loci-controlling quality.

## Experimental procedures

### Laboratory chemicals and reagents

Chemicals and reagents were supplied by Fisher Scientific (Fisher Scientific UK Ltd, Loughborough, UK) or Sigma-Aldrich (Sigma-Aldrich Corporation, St. Louis, MO) unless otherwise stated.

### Primers and DNA

Primers were obtained from Eurofins MWG Operon (Ebersberg, Germany). The dNTPs used in PCR were obtained from Promega (Promega UK Ltd, Southampton, UK). Primers and dNTPs were diluted as in the manufacturer's instructions and stored at −20 °C.

### Polymerase chain reaction (PCR)

PCR was originally described by Saiki *et al*. (1988). PCRs were performed using an Eppendorf Mastercycler PCR machine. Each reaction tube contained a total reaction mixture of 20 μL composed of 12.8 μL H2O, 2 μL 10× PCR buffer, 0.6 μL 50 mm MgCl2, 1.6 μL 2.5 mm dNTPs, 0.4 μL forwards primer, 0.4 μL reverse primer, 2 μL DNA template and 0.2 μL Taq polymerase. PCR buffer and MgCl2 solutions were supplied by Bioline (Bioline Reagents Ltd., London, UK). Unless otherwise stated, the DNA template used in PCR was from a sample of wheat DNA extracted from leaf tissue of Cadenza variety wheat. The general PCR programme used was 94 °C for 5 min, followed by a denaturing, annealing and amplification cycle (repeated 35 times) of 94, 60 (for nongradient PCR) and 72 °C. Once complete, this repeated cycle was followed by 72 °C for 10 min prior to removal of tubes from machine and storage at 4 °C.

### Agarose gel

Electrophoresis gels were 2% molecular biology grade agarose (Bio-Rad Laboratories Inc., Hercules, CA), made up with 0.5× TBE buffer (45 mm Tris base, 45 mm boric acid, 1 mm EDTA). 0.01% v/v 10 mg/mL ethidium bromide was mixed into the buffer once the agarose had been dissolved by heating in a microwave oven and the liquid had begun to cool. Gels were left to solidify in a microgel former with appropriate combs before being placed in a microgel bath and submerged in 0.5× TBE buffer. Next, 2 μL of loading buffer (0.25% w/v xylene cyanol, 0.25% w/v bromophenol blue, 30% v/v glycerol) was added to 10 μL of PCR product in each of the experiment wells (mixed before loading), and 5 μL of DNA ladder was added to each marker well. Once PCR products and marker had been loaded, gels were run at 10 V/cm and then photographed under ultraviolet light.

### Plasmid miniprep

Plasmid was recovered from transformed *Escherichia coli* culture using a Fermentas GeneJET™ plasmid miniprep kit (Fermentas GmbH, St. Leon-Rot, Germany) following the manufacturer's instructions.

### *E. coli* transformation and culture

Competent DH5-α *E. coli* cells were transformed with pHMW-Adh-Nos vector using the method described by Sambrook *et al*. (1989).

### Spectrophotometric analysis of nucleic acids

A NanoDrop 1000 machine (NanoDrop products, Wilmington, DE) was used to quantify DNA and RNA samples using the manufacturer's instructions.

### DNA sequencing

DNA samples were sequenced by Eurofins MWG Operon. DNA samples were prepared for sequencing by following the instructions provided.

### Total RNA extraction from developing wheat endosperm

Total RNA was extracted from wheat endosperm tissue using the method described by Chang *et al*. (1993).

### cDNA synthesis using random hexamer primers

Random hexamer primers were used to synthesize cDNA from total RNA using the method described by Parr *et al*. (1992). Random hexamer primers, RT enzyme, buffer, RNasin RNase inhibitor and dNTPs were supplied by Promega.

### Quantitative reverse transcriptase polymerase chain reaction (qRT-PCR)

Real-time quantitative PCR was first described by Heid *et al*. (1996). qPCR was carried out using 384-well plates in a Roche LightCycler 480 qPCR machine. SYBR Green I master mix was used, and manufacturer's instructions followed for plate preparation. Programme was 95 °C for 5 min; 45 cycles of 95 °C for 10 s, 60 °C for 15 s and 72 °C for 15 s; 95 °C for 5 s, 65 °C for 1 min, slow ramp to 97 and 40 °C for 10 s.

### Phenetic analysis

Phenetic analyses were carried out using *Rab* coding sequence (CDS) data that had been obtained by BLAST or string searches of the NCBI nucleotide and EST databases, as well as Rab protein sequences that had been obtained by following links from corresponding entries in the NCBI nucleotide database or by translating from a CDS that had been assembled from ESTs. The plant species included were *Arabidopsis thaliana*, *Oryza sativa*, *Brachypodium distachyon*, *Triticum aestivum* and *Nicotiana tabacum*. Alignments and phenetic tree files for rectangular trees were generated using MAFFT version 6 online (Katoh and Toh, 2010). Alignments and phenograms for circular trees were produced in MEGA 5 (Tamura *et al*., 2011). All trees were neighbour-joining with 1000 bootstrap replicates, shown on branches as percentage confidence values (Felsenstein, 1985; Saitou and Nei, 1987). Branches corresponding to partitions reproduced in <50% bootstrap replicates were collapsed. The trees were drawn to scale, with branch lengths in the same units as those of the evolutionary distances used to infer the phenetic tree. The evolutionary distances were computed using the Poisson correction method and are in the units of the number of amino acid substitutions per site.

### Cloning of RNAi construct

Bovine serum albumin, calf intestinal alkaline phosphatase, T4 DNA ligase enzyme and *Bam*HI and *Bgl*II restriction enzymes were acquired from Promega. A 270-bp section of the CDS of wheat *RabD2a* gene *Ta.54382* was obtained by PCR using primers with *Bam*HI and *Bgl*II restriction sites added to their 5′ ends (Table 3, Figure S7). The PCR product was purified and cloned into the vector pHMW-Adh-Nos (Nemeth *et al*., 2010; Figure S1). Digestion and ligation of insert and vector were performed using the supplier's instructions. Negative controls used were no enzyme digest (for both insert and vector digests), no enzyme ligation, no vector ligation and no insert ligation. Competent DH5-α *E. coli* cells were used for transformation. *E. coli* cells were grown in LB+ampicillin medium (Per plate: 10 mL sterile LB medium mixed with 10 μL 100 mg/mL ampicillin).

### Wheat transformation and plant growth conditions

Biolistic transformation of wheat (*Triticum aestivum* var. Cadenza) embryos and regeneration of T0 plants were carried out as in Jones and Sparks (2009). Transgenic lines and control plants were grown in the same glasshouse compartments, which were certified for contained-use GMOs. For production of plants, T0 seedlings or seeds were potted in soil and grown in the glasshouse with 18–20 °C day and 14–16 °C night temperatures with a 16-h photoperiod provided by natural light supplemented with banks of Son-T 400 W sodium lamps (Osram Limited, Langley, UK) giving 400–1,000 μmol/m^2^/s PAR. Soil composition contained 75% fine-grade peat, 12% screened sterilized loam, 10% 6-mm screened lime-free grit, 3% medium vermiculite, 2 kg osmocote plus/m^3^ (slow-release fertilizer, 15N/11P/13K plus micronutrients) and 0.5 kg PG mix/m^3^ (14N/16P/18K granular fertilizer plus micronutrients; Petersfield Products, Leicestershire, UK).

### Sodium dodecyl sulphate polyacrylamide gel electrophoresis (SDS-PAGE) of T2 seeds

Three mature seeds from each line to be tested were placed in separate folded pieces of paper and crushed using a pestle. One line per gel was a control line. A 10 mg sample from each crushed seed was weighed out into a 1.5-mL microcentrifuge tube, and a modified version of the gel manufacturer's instructions was followed. Gels were preformed 15-well Bis-Tris 10% acrylamide gels (Invitrogen, Life Technologies Ltd, Paisley, UK), buffer was MOPS SDS running buffer (Invitrogen), gel equipment was a mini dual vertical electrophoresis system (Sigma-Aldrich), and stain was colloidal Coomassie blue stain (Sigma-Aldrich). Phoretix 1D software by Totallab was used for densitometry analysis at Campden BRI.

### Grain size and shape analysis

Mature wheat grains were analysed for grain size and shape using DigiEye apparatus (VeriVide Limited, Enderby, Leicester, LE19 4SG, UK).

### Measurement of flour protein content

Protein content was determined by Dumas combustion in an oxygen-rich environment at high temperatures. Protein is calculated using a factor of 5.7 from total nitrogen determination. Combustion was carried out in a Leco FP528 at Campden BRI.

### Size exclusion high-performance light chromatography (SE-HPLC)

SE-HPLC was carried out on flour samples using the Profilblé method developed jointly by ARVALIS—Institut du vegetal (France) and l'Institut National de la Recherche Agronomique (INRA; Morel *et al*., 2000).

### Dough rheology

Brabender farinograph, extensograph and Reomixer tests were carried out using the manufacturer's instructions. Reomixer traces were converted to PCA components according to the method of Anderson (2004). This uses equations determined at Campden BRI. The smoothed standard-dough traces are converted into two numbers (principal component scores) that are used to plot the position on a quality map, which summarizes the trace characteristics of varying dough development, peak time, peak height and differing breakdown.

## Supporting information

**Figure S1** Vector map of pHMW-Adh-Nos (Nemeth *et al*., 2010). **Figure S2** Size and shape analysis of grain from T2 lines. **Figure S3** Mean size and shape values of grain from T3 lines from a 2D perspective. **Figure S4** Protein content of flour from 20 shortlisted T2 lines as a percentage of dry weight. **Figure S5** SDS-PAGE of mature T1 seeds. **Figure S6** SDS-PAGE of mature T2 seeds. **Figure S7** The Ta.54382 (wheat RabD2a) coding sequence showing selected target region
