# Supplementary material for: Gluten quality of bread wheat is associated with activity of RabD GTPases
Source: Plant Biotechnol J. 2014 Jul 22;13(2):163–76. doi: 10.1111/pbi.12231 (PMC4345403; doi:10.1111/pbi.12231)
Supplement: Supplementary file 1 [file pbi0013-0163-sd1.pdf]

Figure S1

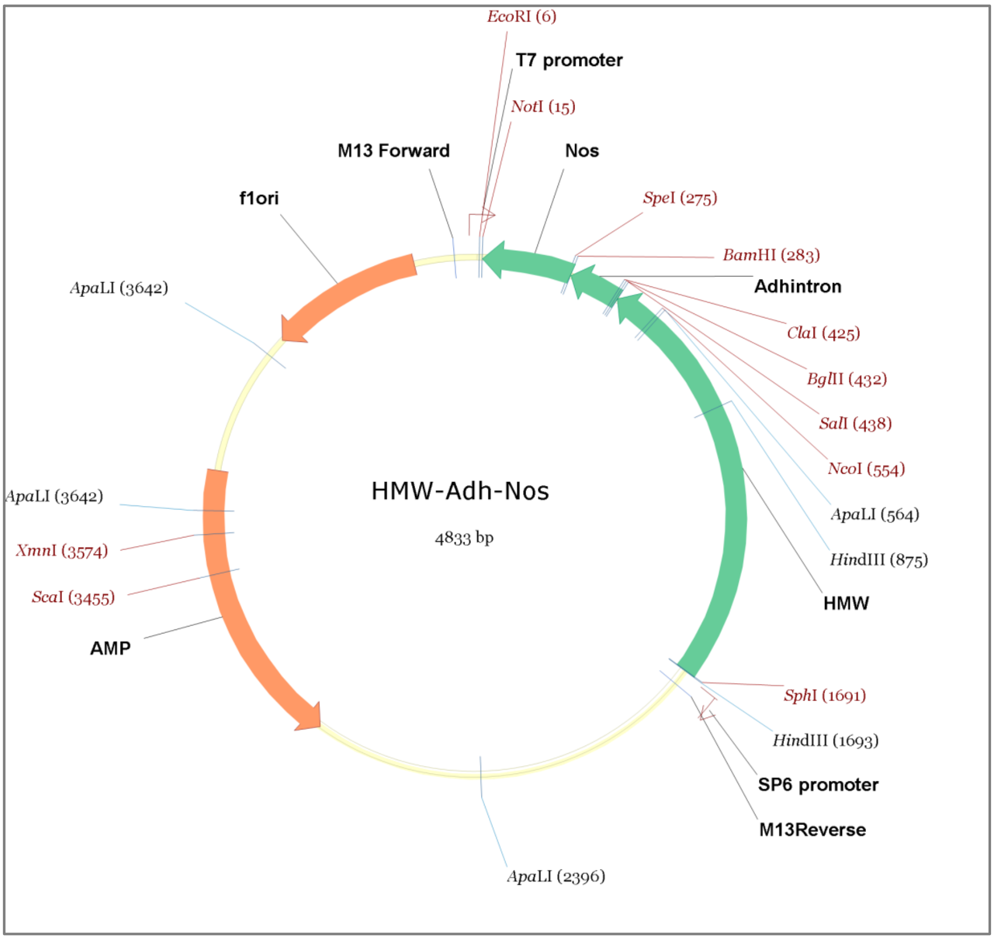

Figure S1 – Vector map of pHMW-Adh-Nos (Nemeth et al, 2010).

Figure S2a

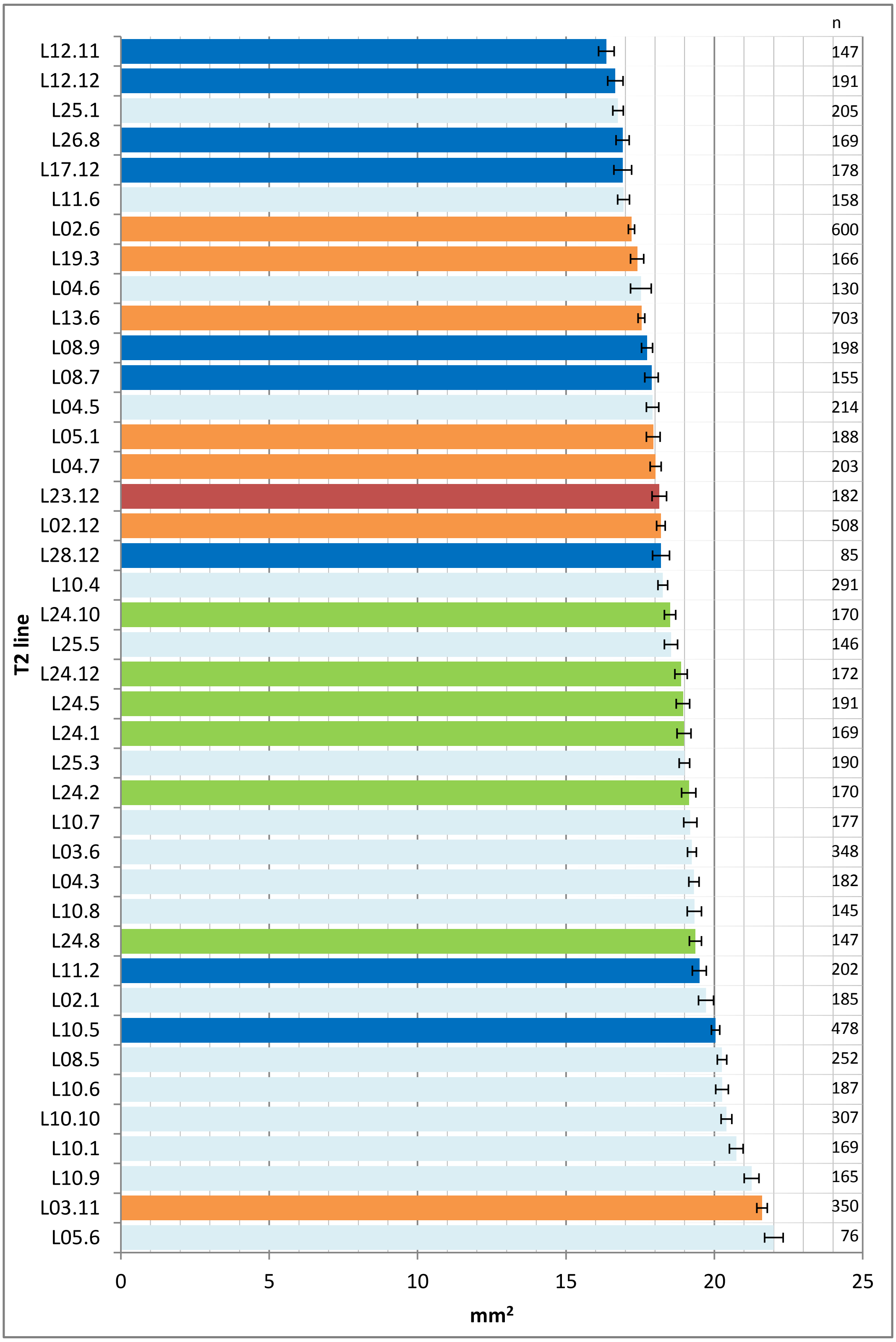

Figure S2a – Plan Area of grain from T2 lines.
Green bars: bar-only control lines, orange bars: appear hemizygous, blue bars: appear homozygous, pale blue bars: zygosity unknown, red bar: appears null segregant. Error bars indicate standard error. Numbers on right (n) indicate number of grains measured for each line.

Figure S2b

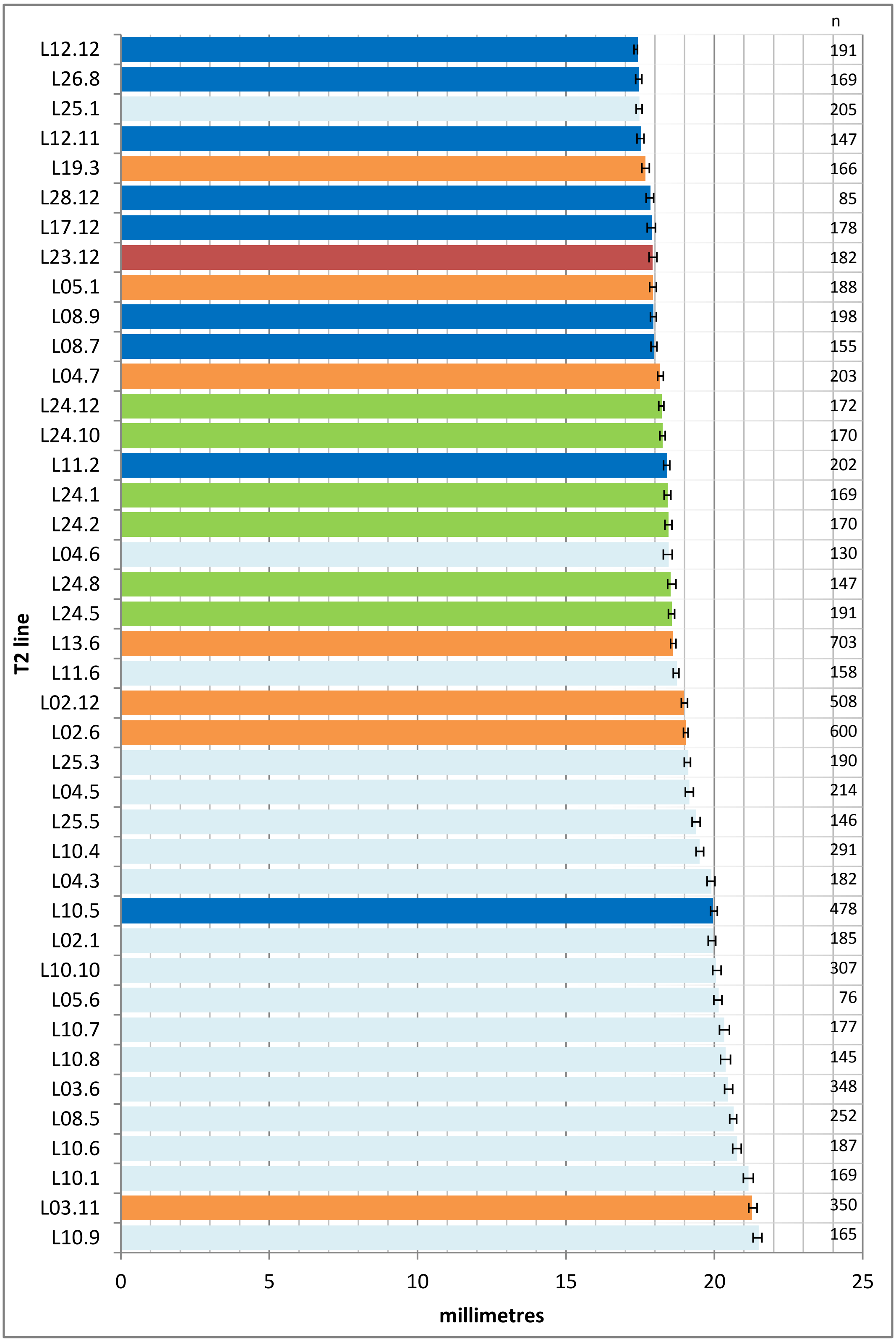

Figure S2b – Perimeter of grain from T2 lines.
Green bars: bar-only control lines, orange bars: appear hemizygous, blue bars: appear homozygous, pale blue bars: zygosity unknown, red bar: appears null segregant. Error bars indicate standard error. Numbers on right (n) indicate number of grains measured for each line.

Figure S2c

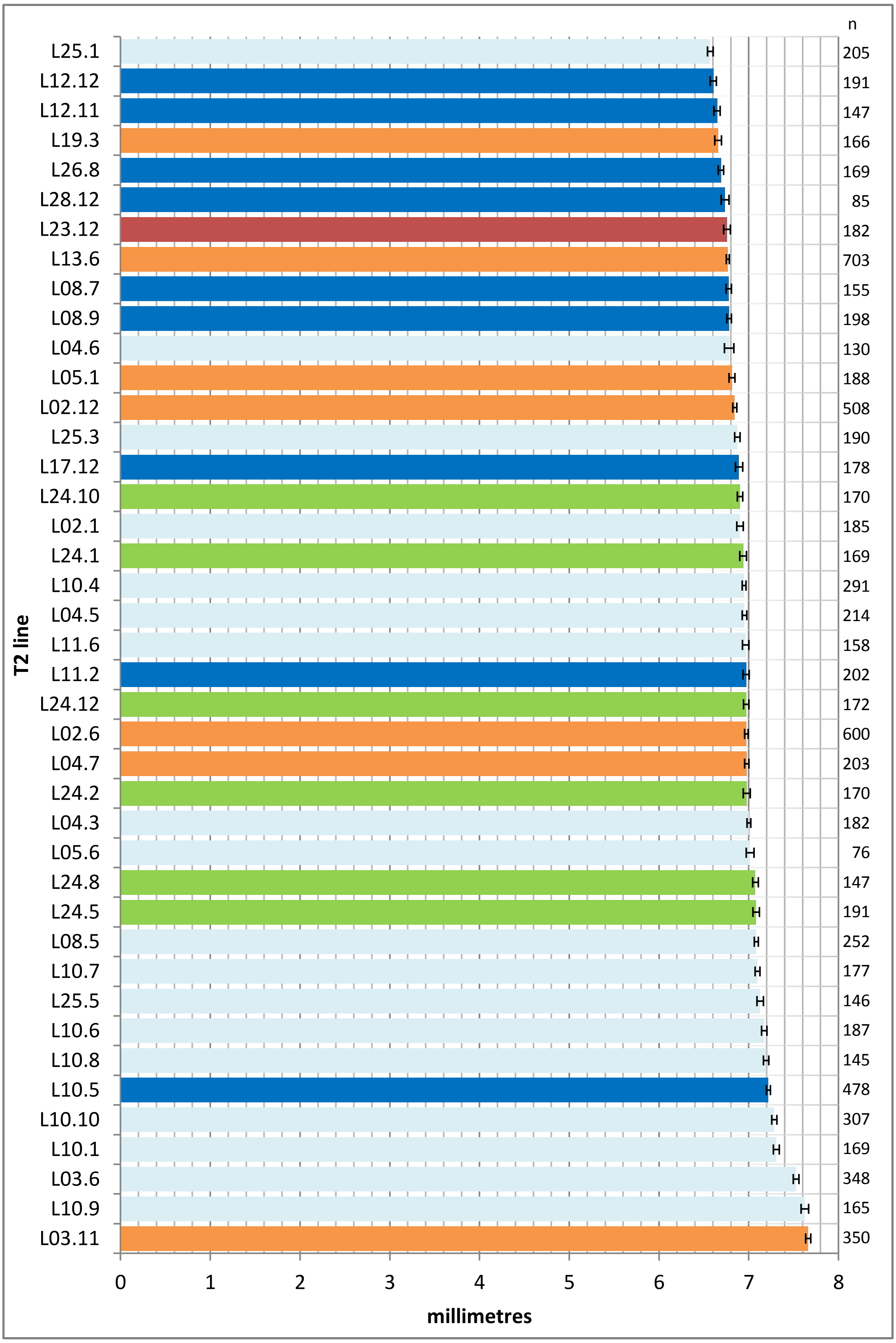

Figure S2c – Length of grain from T2 lines.
Green bars: bar-only control lines, orange bars: appear hemizygous, blue bars: appear homozygous, pale blue bars: zygosity unknown, red bar: appears null segregant. Error bars indicate standard error. Numbers on right (n) indicate number of grains measured for each line.

Figure S2d

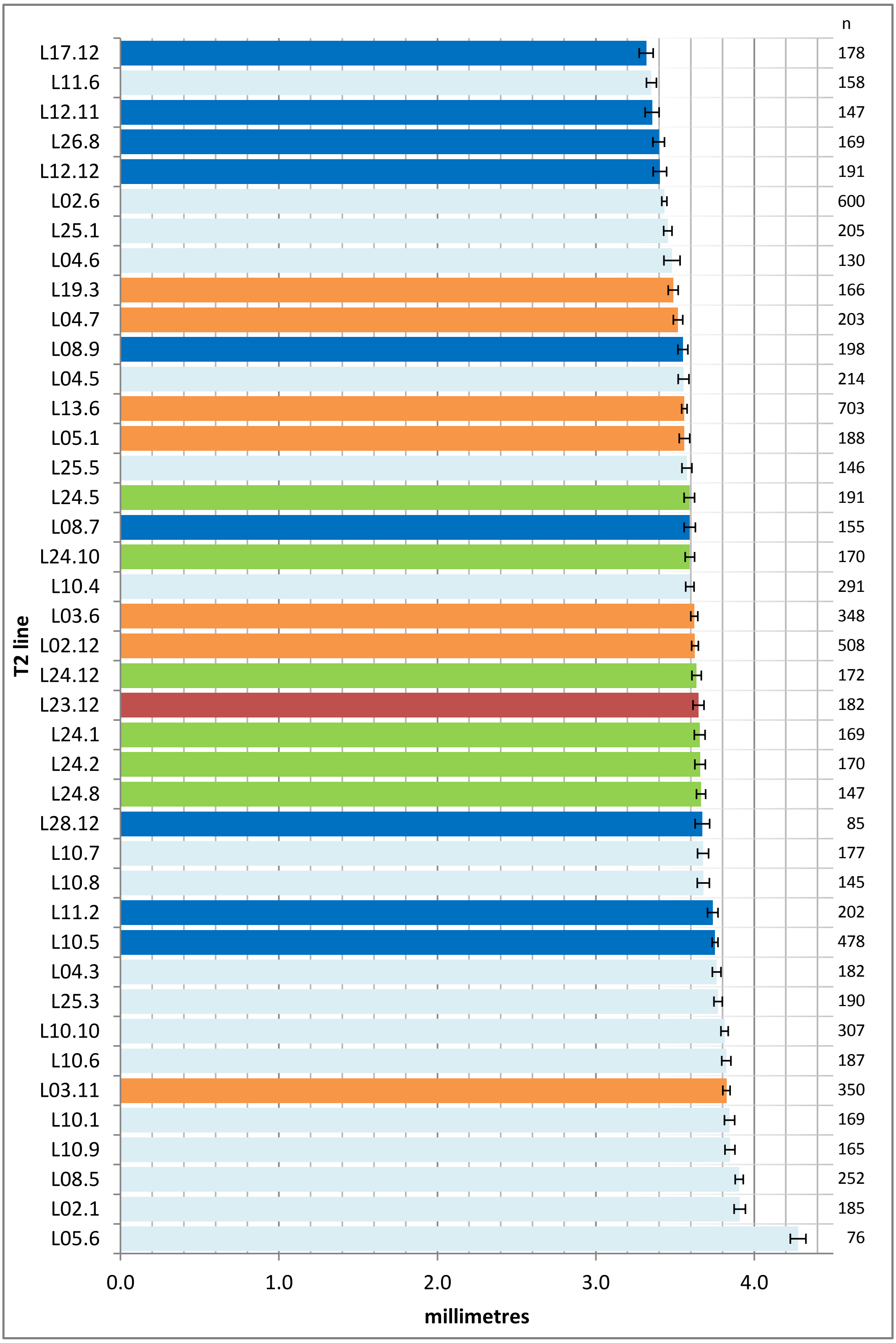

Figure S2d – Width of grain from T2 lines.
Green bars: bar-only control lines, orange bars: appear hemizygous, blue bars: appear homozygous, pale blue bars: zygosity unknown, red bar: appears null segregant. Error bars indicate standard error. Numbers on right (n) indicate number of grains measured for each line.

Figure S2e

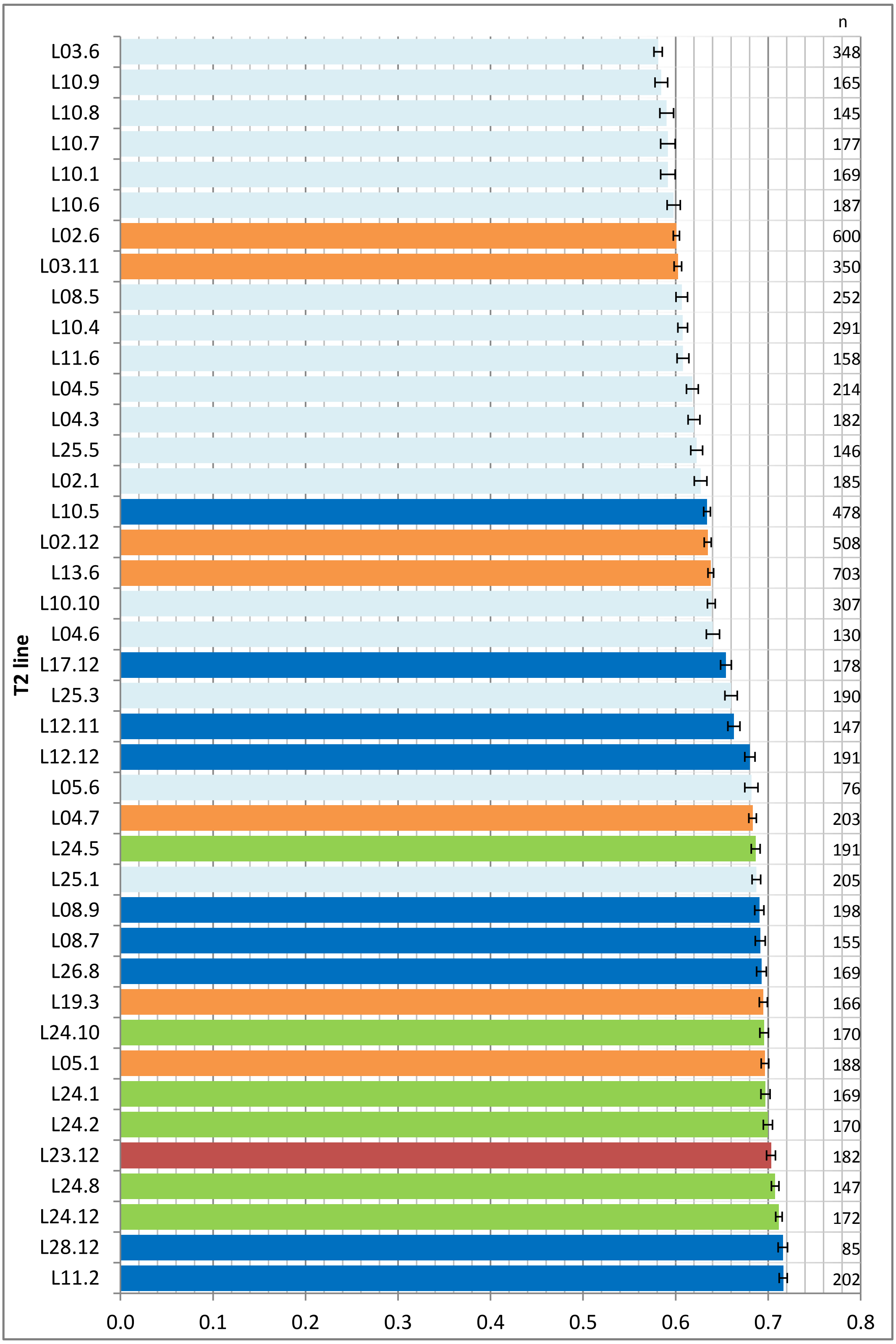

Figure S2e – Circularity of grain from T2 lines.
Green bars: bar-only control lines, orange bars: appear hemizygous, blue bars: appear homozygous, pale blue bars: zygosity unknown, red bar: appears null segregant. Error bars indicate standard error. Numbers on right (n) indicate number of grains measured for each line. Circularity = 4π × Plan Area/Perimeter^2. A circularity of 1 indicates a perfect circle and increasingly elongated shapes give values approaching 0.

Figure S3

(a)

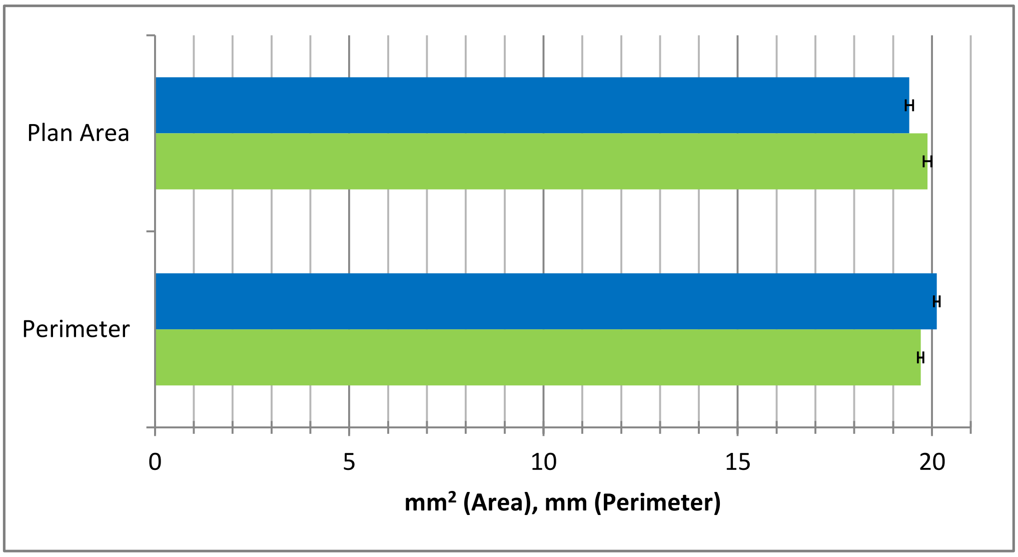

(b)

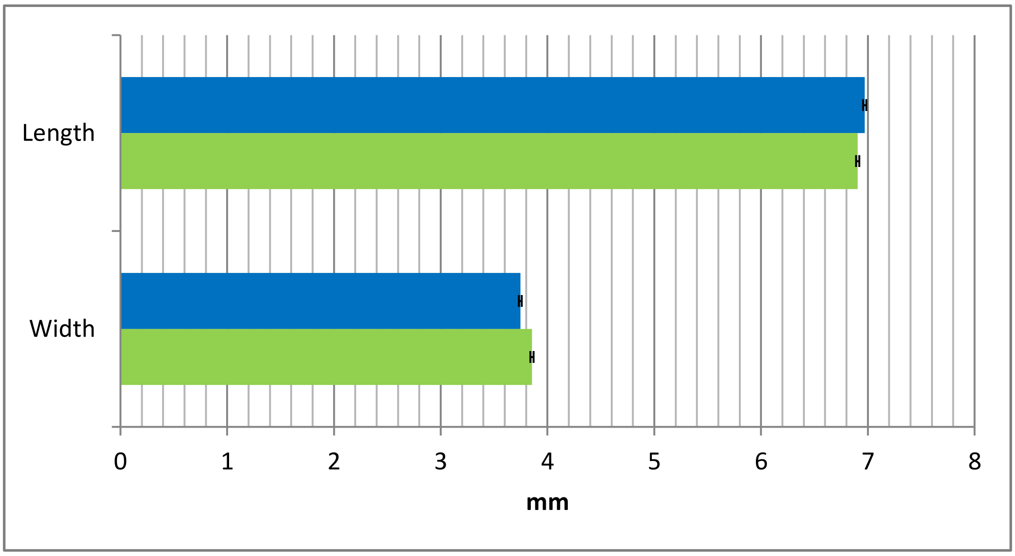

(c)

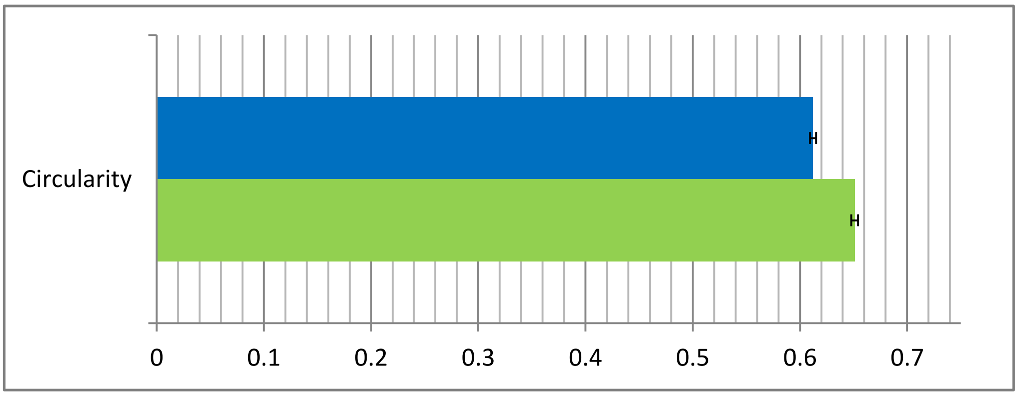

Figure S3 – Mean size and shape values of grain from T3 lines from a 2D perspective.
(a): Plan Area and Perimeter; (b): Length and Width; (c): Circularity. Blue bars indicate T3 knockdown line L10.5, green bars indicate T3 bar-only control line L24.2. Error bars indicate standard error.

Figure S4

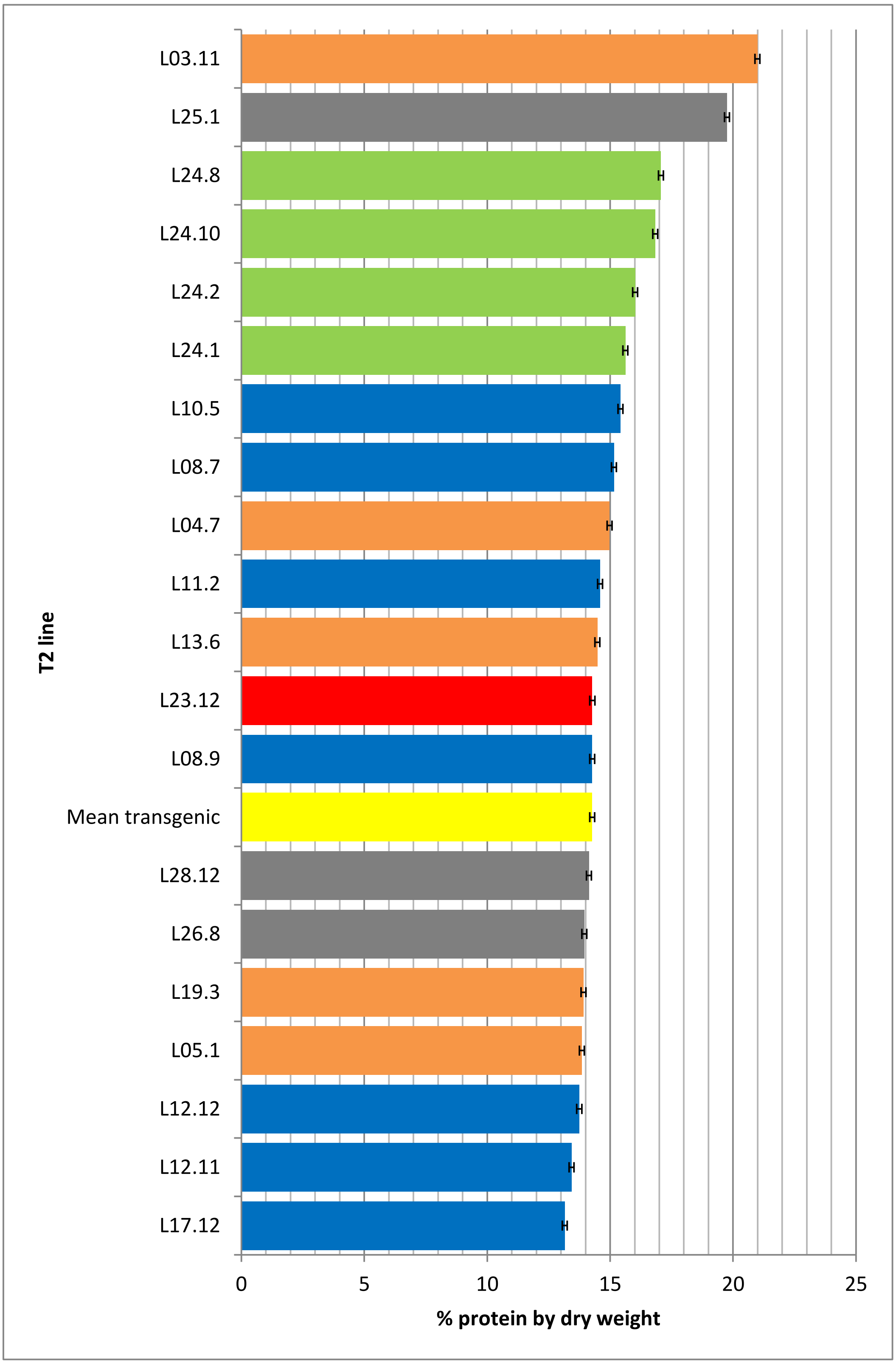

Figure S4 – Protein content of flour from 20 shortlisted T2 lines as a percentage of dry weight.
Green bars: bar-only control lines; orange bars: appear hemizygous; blue bars: appear homozygous; grey bars: zygosity unknown; red bar: appears null segregant; yellow bar: mean of transgenic lines except L03.11. Error bars indicate predicted error of protein and moisture measuring techniques (0.1).

Figure S5

(a)

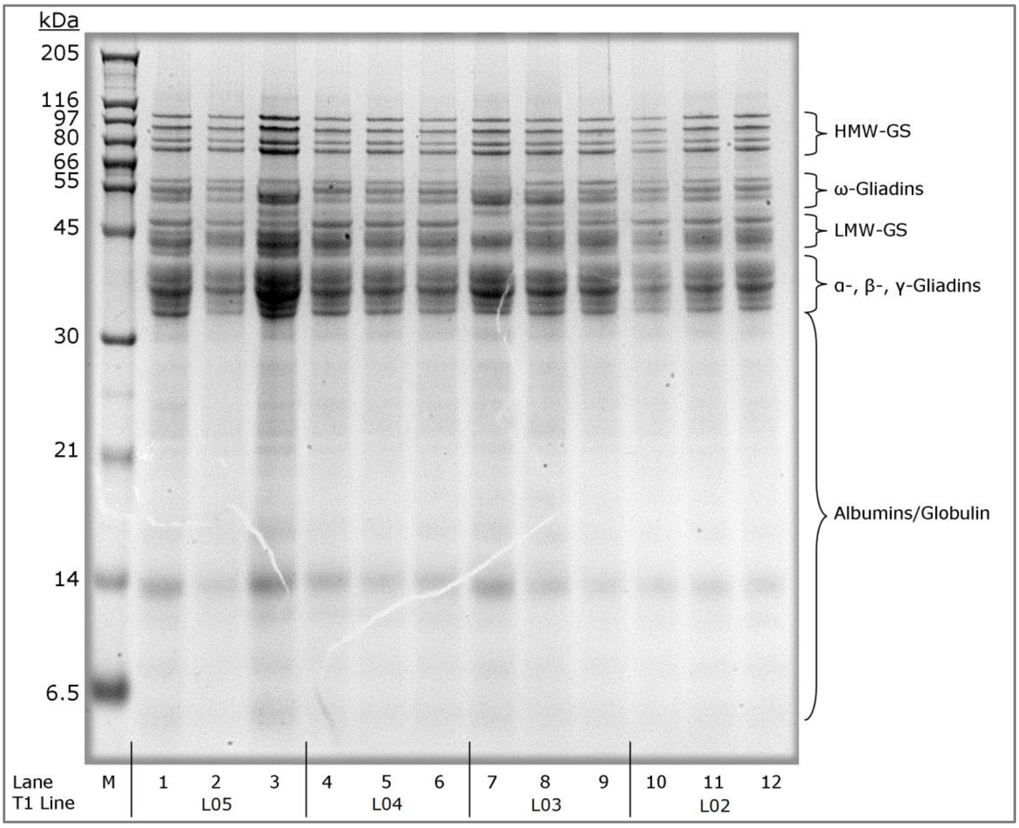

(b)

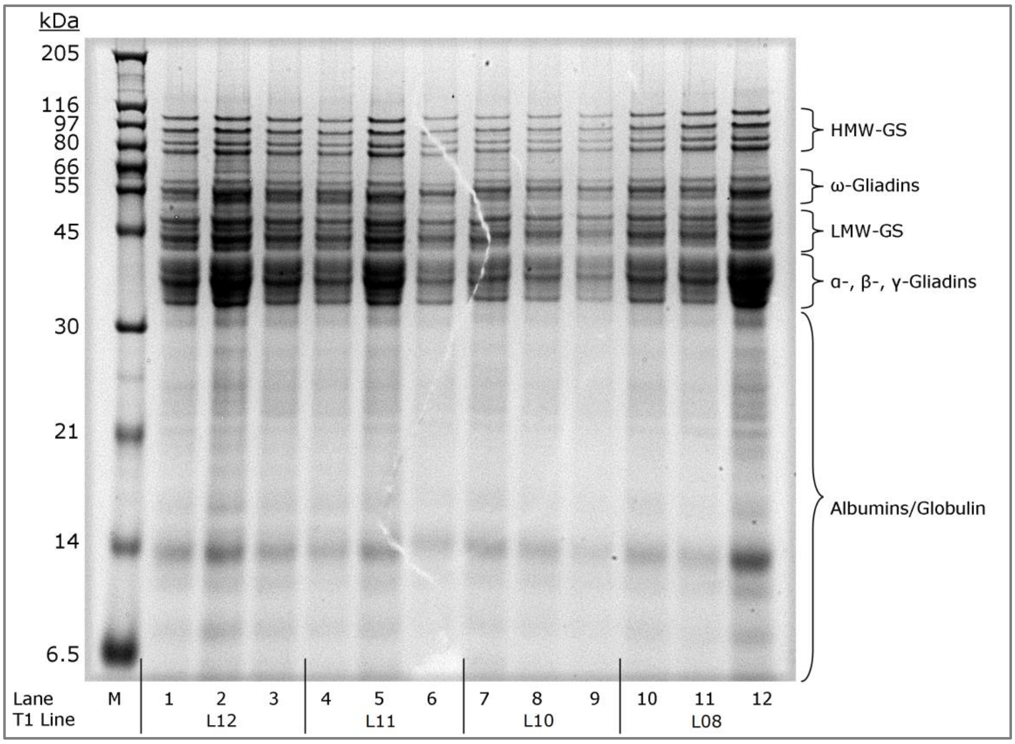

Figure S5 – SDS-PAGE of mature T1 seeds.
Sizes of marker proteins are indicated on the left in kDa. Lane numbers and T1 lines are displayed below the gels. Categories of seed storage proteins are indicated on the right. M indicates a lane containing protein marker.

Figure S6a,b

(a)

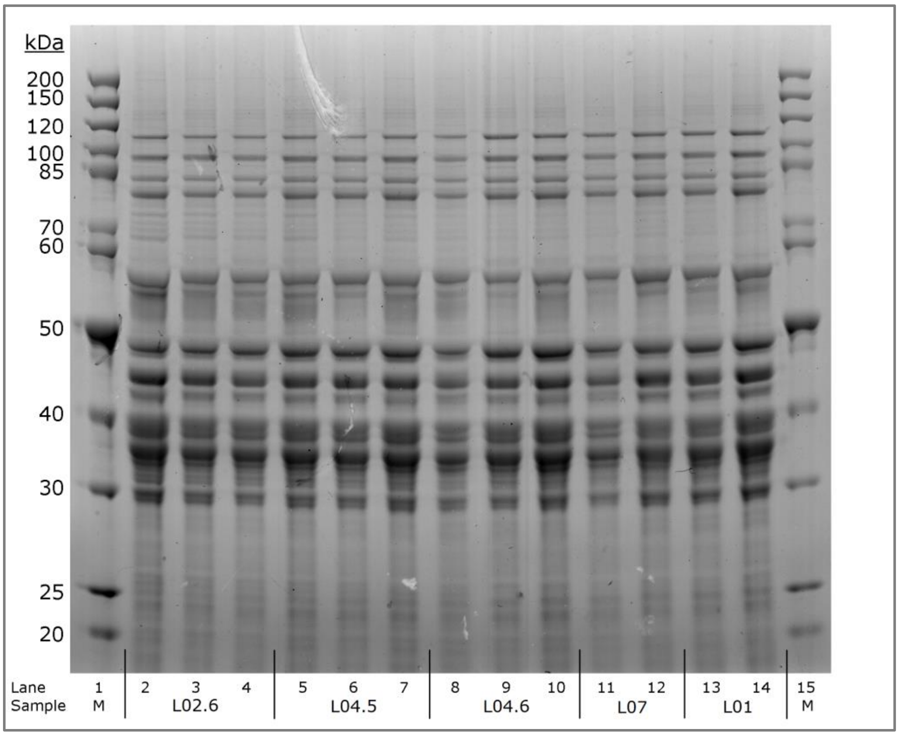

(b)

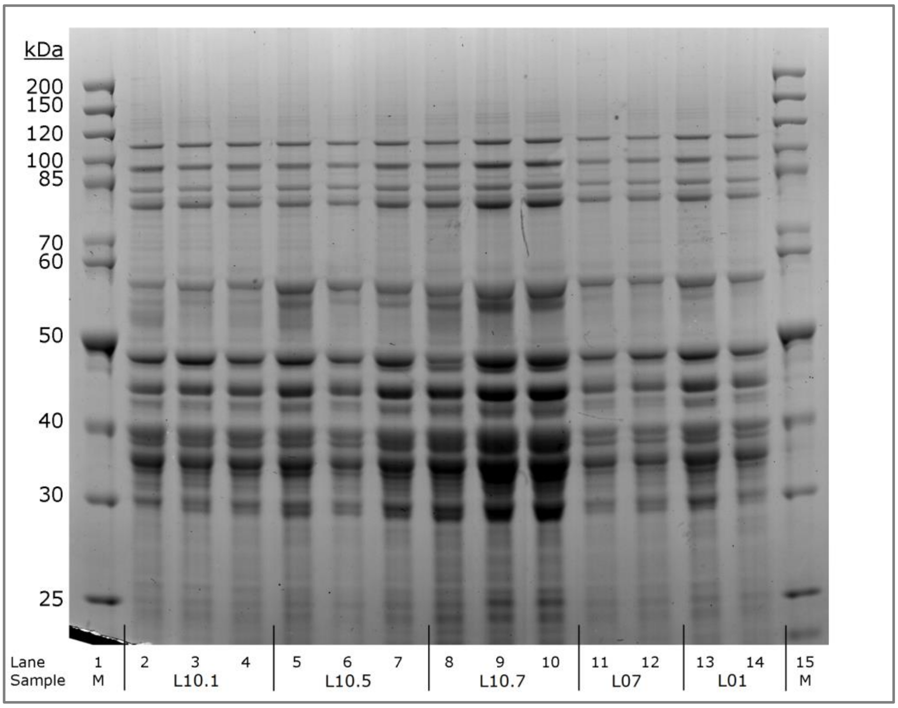

Figure S6a,b – SDS-PAGE of mature T2 seeds.
Sizes of marker proteins are indicated on the left in kDa. Lane numbers and line names are displayed below the gels. L07 = T1 bar-only control line, L01 = T1 gold-only control line. M indicates a lane containing protein marker.

Figure S6c,d

(c)

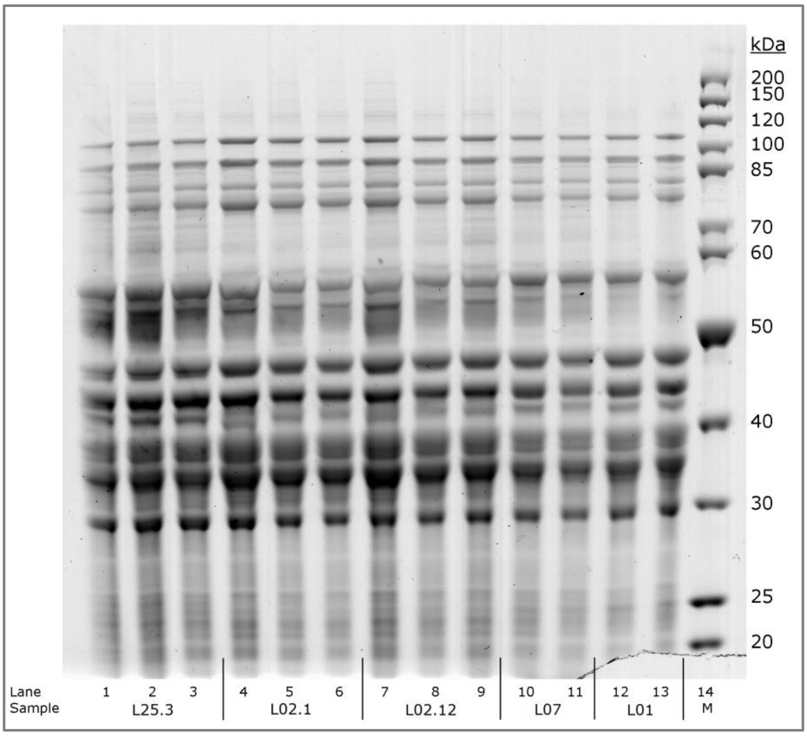

(d)

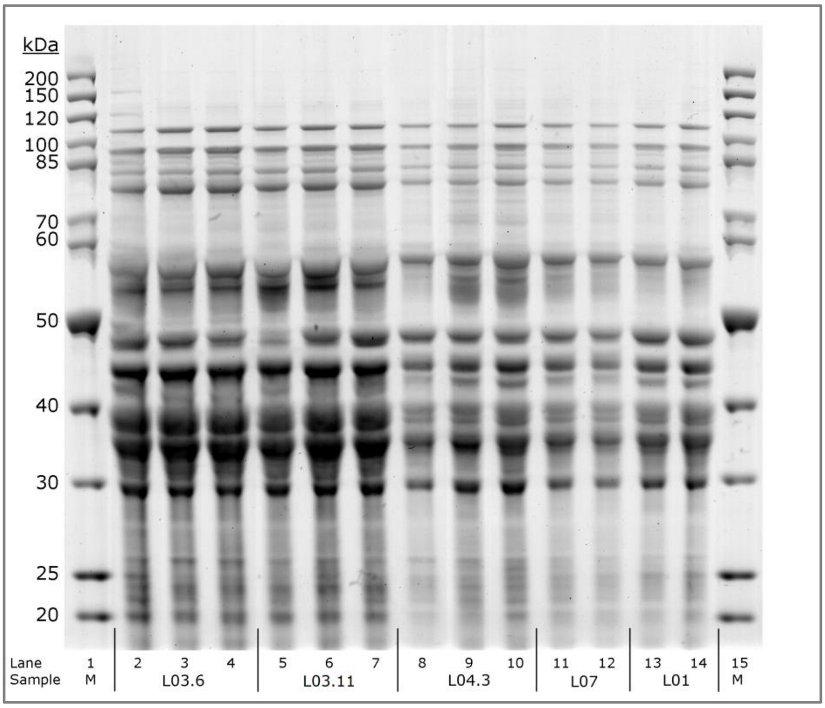

Figure S6c,d – SDS-PAGE of mature T2 seeds.
Sizes of marker proteins are indicated on the right/left in kDa. Lane numbers and line names are displayed below the gels. L07 = T1 bar-only control line, L01 = T1 gold-only control line. M indicates a lane containing protein marker.

Figure S6e

(e)

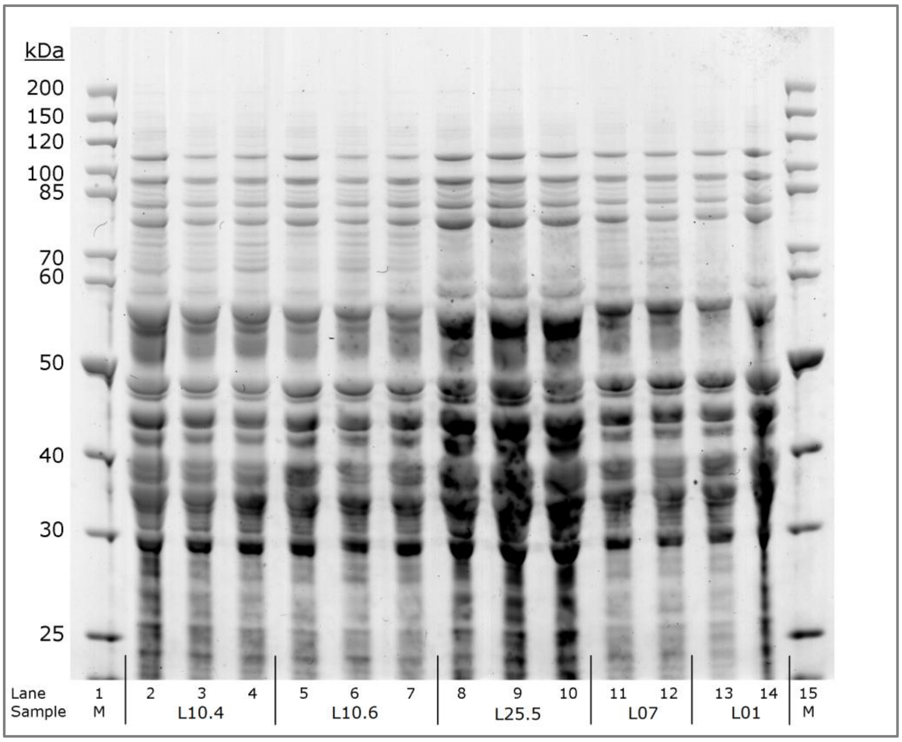

Figure S6e – SDS-PAGE of mature T2 seeds.
Sizes of marker proteins are indicated on the right in kDa. Lane numbers and line names are displayed below the gels. L07 = T1 bar-only control line, L01 = T1 gold-only control line. M indicates a lane containing protein marker.

Figure S6f,g

(f)

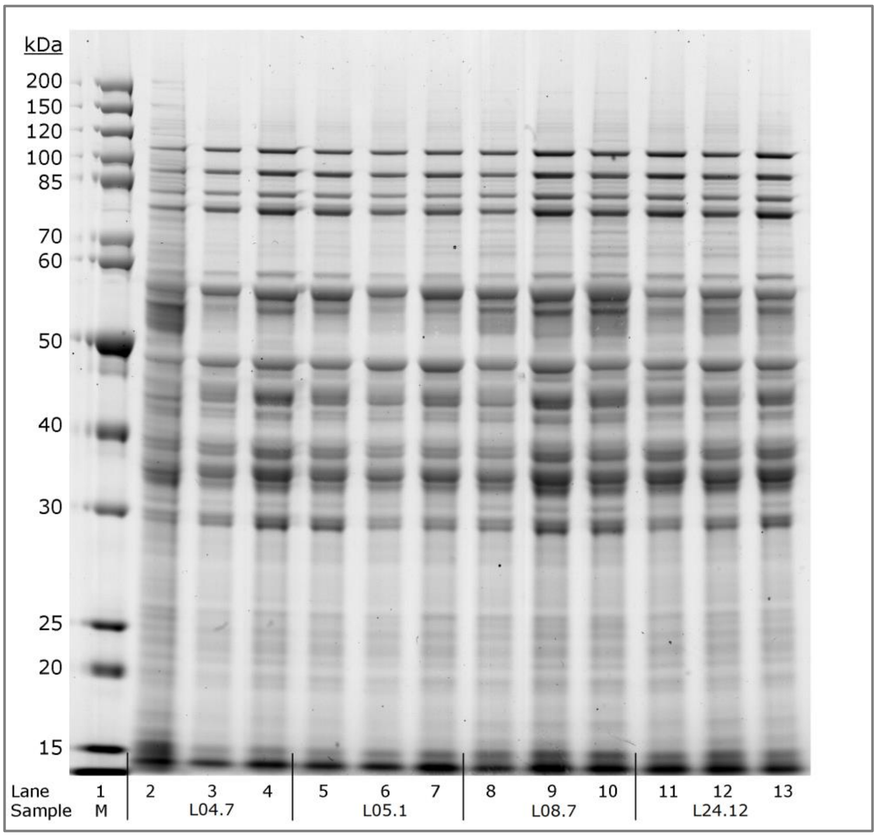

(g)

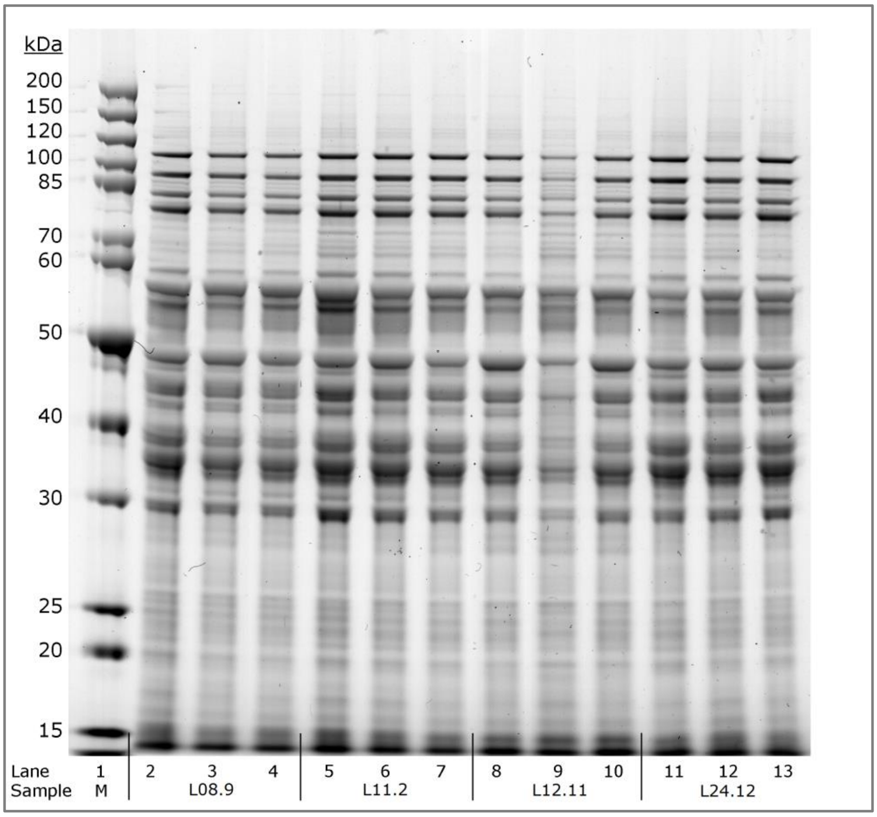

Figure S6f,g – SDS-PAGE of mature T2 seeds.
Sizes of marker proteins are indicated on the left in kDa. Lane numbers and line names are displayed below the gels. L24.12 = T2 bar-only control line. M indicates a lane containing protein marker.

Figure S6h,i

(h)

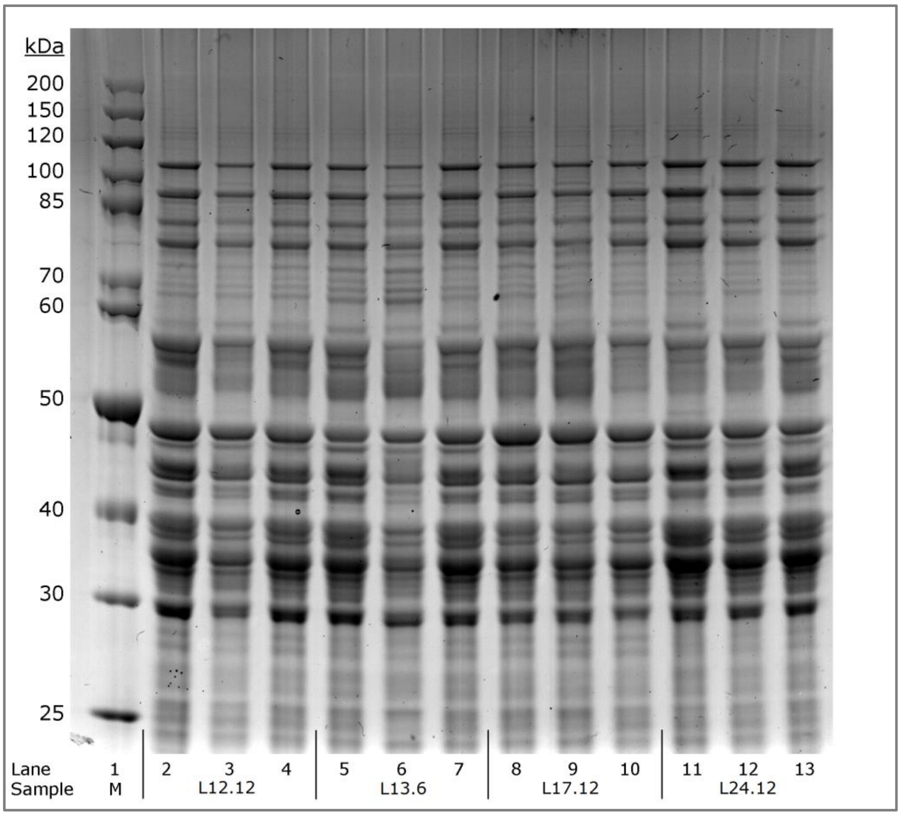

(i)

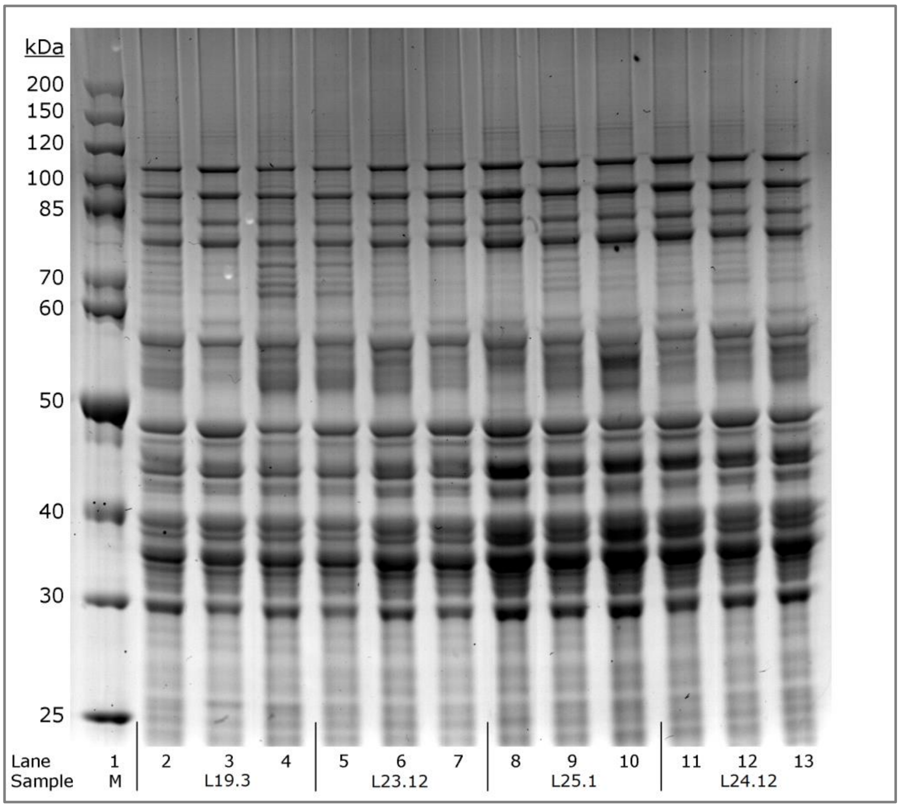

Figure S6h,i – SDS-PAGE of mature T2 seeds.
Sizes of marker proteins are indicated on the left in kDa. Lane numbers and line names are displayed below the gels. L24.12 = T2 bar-only control line. M indicates a lane containing protein marker.

Figure S6j

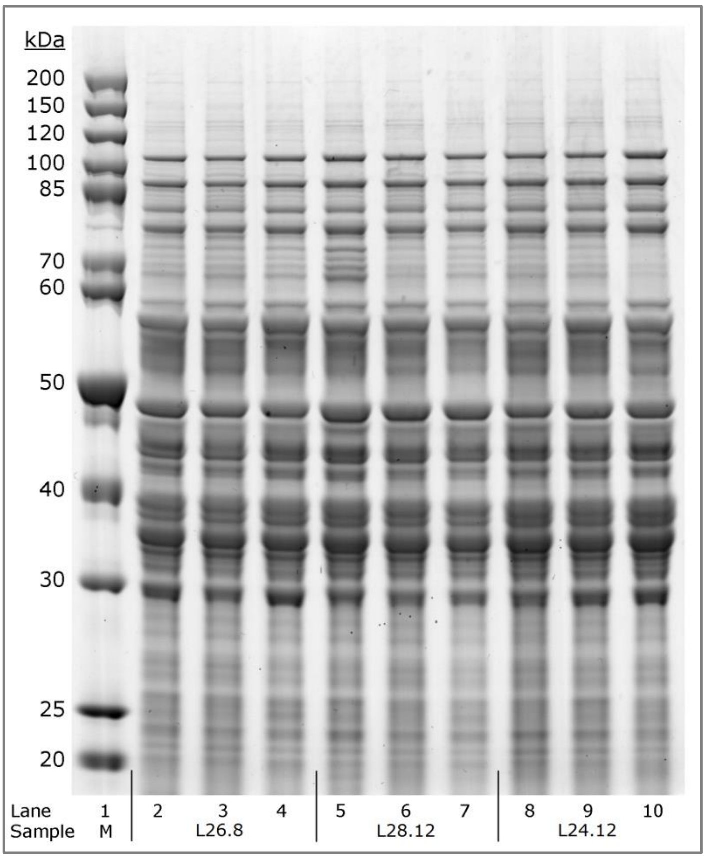

Figure S6j – SDS-PAGE of mature T2 seeds.
Sizes of marker proteins are indicated on the left in kDa. Lane numbers and line names are displayed below the gels. L24.12 = T2 bar-only control line. M indicates a lane containing protein marker.

Figure S7

```
ATGAATCCGGAGTATGACTATCTCTTTAAGCTTCTGCTTATCGGAGACTC 50
AGGTGTTGGCAAGTCATGCCTTCTCTTAAGATTTGCCGATGACTCGTACC 100
TGGAGAGTTATATCAGTACTATTGGTGTTGATTTCAAAATACGTACCGTG 150
GAGCAAGATGGAAAGACTATGAAGCTGCAAATCTGGGACACTGCTGGGCA 200
AGAACGCTTCAGAACTATTACTAGCAGCTACTATCGAGGGGCTCACGGGA 250
TCATTATTGTCTATGACGTGACAGACCAGGACAGCTTCAACAATGTGAAG 300
CAGTGGTTGAACGAGATTGATCGCTATGCTAGTGAGAATGTTAACAAGCT 350
TCTTGTAGGGAACAAATCTGATCTCACTGACAAAAGAGTTGTATCATATG 400
AGACAGCGAAGGCATTTGCTGATGAGATTGGCATCCCATTCATGGAGACC 450
AGTGCAAAGAATGCCTTGAACGTTGAGCAGGCTTTCATGGCTATGTCTGC 500
TTCAATCAAGGACAGGATGGCGAGCCAGCCAGCCGCAAACAGCGCTCGCC 550
CAGCCACGGTGCAGATCCGCGGGCAACCTGTCGAACAGAAGACGAGCTGC 600
TGCTCTTCT 609
```

Figure S7 – The Ta.54382 (wheat RabD2a) coding sequence showing selected target region.
The 270bp target region is highlighted in yellow, with forward and reverse primer regions shown in green and cyan respectively. The 23nt match with Ta.54881 (RabD2b) is highlighted in dark blue.
